# Construction of angstrom-scale ion channels with versatile pore configurations and sizes by metal-organic frameworks

**DOI:** 10.1038/s41467-023-35970-x

**Published:** 2023-01-18

**Authors:** Xingya Li, Gengping Jiang, Meipeng Jian, Chen Zhao, Jue Hou, Aaron W. Thornton, Xinyi Zhang, Jefferson Zhe Liu, Benny D. Freeman, Huanting Wang, Lei Jiang, Huacheng Zhang

**Affiliations:** 1 Department of Chemical and Biological Engineering, Monash University, Clayton, VIC 3800 Australia; 2 College of Science, Wuhan University of Science and Technology, Wuhan, 430072 China; 3 Chemical and Environmental Engineering, School of Engineering, RMIT University, Melbourne, VIC 3000 Australia; 4 Manufacturing, CSIRO, Clayton, VIC 3168 Australia; 5 Hubei Key Laboratory of Ferro- & Piezoelectric Materials and Devices, Faculty of Physics & Electronic Science, Hubei University, Wuhan, 430062 China; 6 Department of Mechanical Engineering, The University of Melbourne, Parkville, VIC 3010 Australia; 7 Department of Chemical Engineering, The University of Texas at Austin, Austin, TX 78712 USA

**Keywords:** Organic-inorganic nanostructures, Organic-inorganic nanostructures, Metal-organic frameworks

## Abstract

Controllable fabrication of angstrom-size channels has been long desired to mimic biological ion channels for the fundamental study of ion transport. Here we report a strategy for fabricating angstrom-scale ion channels with one-dimensional (1D) to three-dimensional (3D) pore structures by the growth of metal-organic frameworks (MOFs) into nanochannels. The 1D MIL-53 channels of flexible pore sizes around 5.2 × 8.9 Å can transport cations rapidly, with one to two orders of magnitude higher conductivities and mobilities than MOF channels of hybrid pore configurations and sizes, including Al-TCPP with 1D ~8 Å channels connected by 2D ~6 Å interlayers, and 3D UiO-66 channels of ~6 Å windows and 9 − 12 Å cavities. Furthermore, the 3D MOF channels exhibit better ion sieving properties than those of 1D and 2D MOF channels. Theoretical simulations reveal that ion transport through 2D and 3D MOF channels should undergo multiple dehydration-rehydration processes, resulting in higher energy barriers than pure 1D channels. These findings offer a platform for studying ion transport properties at angstrom-scale confinement and provide guidelines for improving the efficiency of ionic separations and nanofluidics.

## Abstract (web summary)

The fabrication of synthetic nanochannels for ion separation is challenging. Here, the authors synthesize angstrom-scale ion channels featuring one-dimensional to three-dimensional pore configurations by growing metal‒organic frameworks into nanochannels; the structure of the pore influences the ion transport performance.

## Introduction

Angstrom-size ion channels with versatile pore structures and tunable functionalities are crucial for developing the high-efficiency, low-energy consumption technologies required for membrane separation, energy conversion and storage applications^1–3^. Many natural ion channels comprise angstrom-scale pore structures with specific sizes and configurations for rapid and selective ion permeation^4,5^. For example, proton (H^+^) channels generally are 3- to 6-Å regions in the centers of channels and confine water molecules to ordered hydrogen-bonded chains for ultrafast proton transport^6–10^. Potassium (K^+^) ion channels, with ~3.5 Å selective filters on one side and ~10 Å cavities on the other side of the channels^11–13^, can transport K^+^ at a rate of 10^8 ^ions s^−1^ with a selectivity up to 10^4^ over sodium (Na^+^)^14,15^. Na^+^ ion channels, comprised of selective filters of 3- to 5-Å and cavities of ~12 Å in diameter, exhibit Na^+^/K^+^ selectivities ranging from 10 to 30 and a high transport rate of 10^6 ^ions s^−1^ of Na^+^. Because of the window-cavity channel structure, the transport rate of cations through Na^+^ channels follows the sequence Li^+^ > Na^+^ > K^+^^16–18^. These ion channels exhibit specific ion perm-selectivity due to the unique configurations and channel sizes at angstrom scale. To fully understand ion transport mechanisms in biological ion channels, we need to construct analogous ion channels with diverse pore configurations and sizes.

One-dimensional (1D) carbon nanotubes (CNTs) of 0.8-nm in diameter have been fabricated for the study of ion transport. The proton conductivity of CNT channels can exceed that of biological proton channels, owing to the sub-1-nm confinement that forces water molecules into a single chain for proton Grotthus conduction^19^. CNTs can also be tuned for cation selective transport while blocking anions by the deprotonated carboxylate groups on the channel wall^20^. Two-dimensional (2D) slit-like channels with a height of ~6.7 Å have been constructed from graphite, hexagonal boron nitride (h-BN), and molybdenum disulfide (MoS_2_). Ions with hydrated diameters larger than the slit height can still be transported through these 2D channels, but with reduced conductivities and mobilities, because of the angstrom size constraint^21^. Narrowing the height of above 2D slits to ~3.4 Å allows only accommodating one monolayer of water molecules. Due to the 2D slit restriction, other hydrated ions can hardly be further compressed to enter the channel, whereas protons can readily diffuse through the 2D monolayered water molecules with relatively low conductivity^22^. Despite having various angstrom-scale pore sizes, the resulting nanotubes and nanoslits can form channels with only 1D or 2D configurations for ion transport in one direction^23–28^, and this channel geometry imposes limitations in the construction of versatile channel structures and the investigation of confined ion transport properties.

Metal-organic frameworks (MOFs) have recently been studied as novel ion transport materials, owing to their permanent channel porosity, adjustable angstrom-scale pore sizes, and diverse channel geometry^29–32^. 3D MOF channels, where ions can transport in three directions, have been frequently investigated. For instance, ZIF-8 channels with window-cavity structures have been prepared for ultrafast and selective transport of alkali metal ions, where the angstrom-size windows serve as ion sieving filters, and the nanometer-size cavities function as ion transport pathways^29^. Zirconium-based MOFs (UiO-66-(COOH)_2_) have been assembled inside a nanometer-size polymer channel to fabricate heterogeneous subnano-to-nano sized channels for rapid and selective transport of alkali metal ions while essentially rejecting divalent cations^31^. Very recently, photo-responsive Al-TCPP channels with 2D configurations have been used to transport ions to accomplish osmotic energy conversion^33^. In addition, unidirectional and selective proton conduction has been achieved in heterogeneous nanochannels based on MIL-derivative 1D MOF channels^34^. Although unique ion transport phenomena have been identified in MOF channels with several configurations, there has been little systematic study of the relationship between ion transport properties and MOF channel geometries and sizes.

Herein, three MOF channels of different pore sizes and configurations are fabricated, including 1D MIL-53 channels with flexible pore sizes between narrow pore (NP, 2.6 Å × 13.6 Å) and large pore (LP, 8.5 Å × 8.5 Å), Al-TCPP with 1D channels of ~8 Å vertically connected by 2D interlayer spaces of ~6 Å, and 3D UiO-66 channels composed of an octahedral cavity of ~12 Å and a tetrahedral cavity of ~9 Å linked by a window aperture of ~6 Å, to investigate the configuration and size effects on ion transport through angstrom-scale MOF channels. In MIL-53 channels, the conductivities and mobilities of monovalent cations are close to those in bulk solution. In Al-TCPP channels, the conductivities and mobilities of cations are one order of magnitude lower than those in 1D MIL-53 channels, in the following sequence: H^+^ > M^+^ (K^+^, Na^+^, Li^+^) >M^2+^ (Ca^2+^, Mg^2+^) >Al^3+^, and the sequence of monovalent cations still follows: K^+^ > Na^+^ > Li^+^, as observed in bulk solution. The conductivities and mobilities of cations in 3D UiO-66 channels are two orders of magnitude lower than those in 1D MIL-53 channels, and the order of conductivities is: H^+^ > M^+^ > M^2+^ > Al^3+^, where there is a reversal in the transport order of monovalent cations, to Li^+^ > Na^+^ > K^+^. According to theoretical simulations and calculations, the energy barriers for ion transport through the pure 1D channels are lower than those through the complex channel connection, e.g., 1D channels vertically connected by 2D interlayers, and 3D channels with window-cavity pore structures.

## Results

### Fabrication of MOF channels with various configurations and sizes

**Fig. 1:**
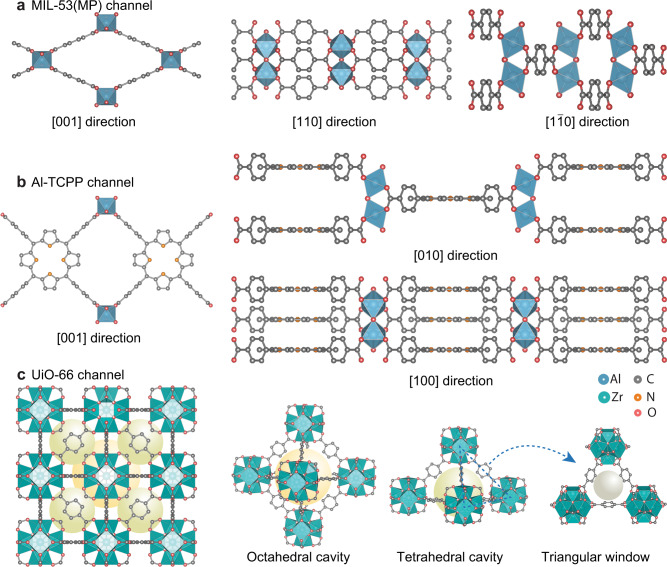
Schematics of MOF channel structures with various configurations (top views). **a** MIL-53(MP) channel with medium pore form along the [001] direction, [110] direction, and [1$\overline{1}$0] direction, respectively. The 1D channel is in the [001] direction. **b** Al-TCPP channel along the [001] direction, [010] direction, and [100] direction, respectively. The 1D channel is in the [001] direction, and the 2D interlayer space is in the [100] direction. **c** UiO-66 with 3D channels of an octahedral cavity, a tetrahedral cavity, and a triangular window, respectively. UiO-66 has the same channel structure in three directions due to the cubic symmetric crystal structure. Accordingly, the complexity degree of MOF channels follows the order: MIL-53(MP) < Al-TCPP < UiO-66.

**Fig. 2:**
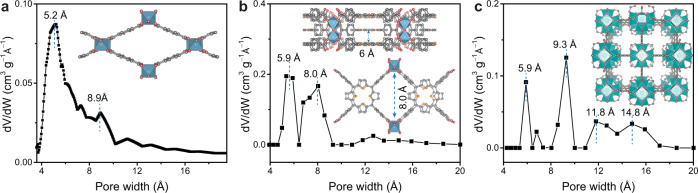
Pore size distributions of MOF channels. **a** The 1D MIL-53(MP) channels of ~5.2 × 8.9 Å in diameter. **b** The Al-TCPP channel composed of ~8 Å 1D channels and ~6 Å 2D interlayer spaces. **c** The 3D porous UiO-66 channel composed of ~12 Å octahedral cavities, ~9 Å tetrahedral cavities, and ~6 Å triangular windows.

MOF channels were fabricated by contra-diffusion growth of MOF crystals into 12-µm-thick single-nanochannel PET membranes (see Methods for more details). A bullet nanochannel embedded within a PET membrane was fabricated via an ion-track-etching method^35^. After contra-diffusion growth of MOFs (MIL-53, Al-TCPP, and UiO-66) (Supplementary Fig. 1), the bullet PET channel was filled with MOF crystals, confirmed by SEM images and energy dispersive X-ray (EDX) spectra (Supplementary Figs. 2–4). The powder X-ray diffraction (XRD) patterns also verified the successful synthesis of MOF crystals and a hybrid pore form of MIL-53 between NP and LP under the experimental conditions (denoted as medium pore (MP), a hypothetical structure representing the mixture of large pore and narrow pore phases), see Supplementary Notes and Supplementary Fig. 5 for more discussion). Fig 1 shows MOF channels with various configurations. Top views of MIL-53(MP) channel^36^ are shown in the [001], [110], and [1$\overline{1}$0] directions, where the 1D channel is along the [001] direction (Fig. 1a). As for Al-TCPP channel (Fig. 1b), top views are shown in the [001], [010], and [100] directions. The 1D channel of Al-TCPP is along the [001] direction, and the 2D interlayer space is along the [100] direction^37^. Top views of UiO-66 with 3D channels are displayed, including an octahedral cavity, a tetrahedral cavity, and a triangular window in Fig. 1c^38^. UiO-66 has the same channel structure in three directions because of the cubic symmetric crystal structure. Accordingly, the three MOF channel configurations exhibit a degree of complexity following the order: MIL-53(MP) < Al-TCPP < UiO-66. Moreover, the pore size distributions calculated from N_2_ adsorption-desorption isotherms show that the 1D channels of MIL-53(MP) have a wider pore size distribution between 4 and 12 Å than MIL-53(LP) (Fig. 2a and Supplementary Fig. 6). Different from MIL-53(LP) of one pore size peak at 8.5 Å, MIL-53(MP) shows two pore size peaks at 5.2 and 8.9 Å, indicating the hybrid pore form of MIL-53 in the electrolyte solution. In contrast, Al-TCPP has a 1D channel of ~8 Å and an interlayer distance of ~6 Å (Fig. 2b), and UiO-66 possesses 3D channels comprised of an octahedral cavity of ~12 Å, a tetrahedral cavity of ~9 Å, and a triangular window of ~6 Å (Fig. 2c).

### Ion transport properties in MOF channels

**Fig. 3:**
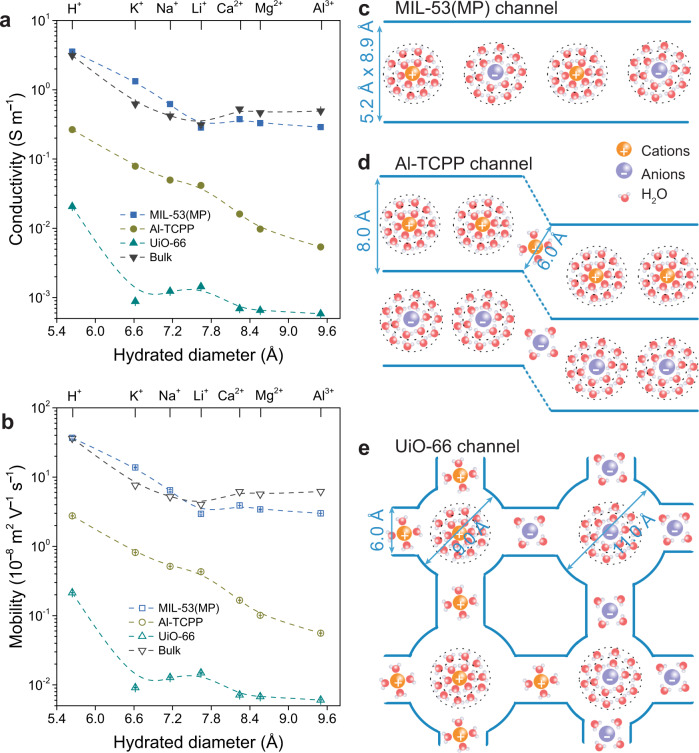
Cations transport properties in MOF channels. **a** Conductivities of cations in MOF and bulk solution. **b** Mobilities of cations in MOF channels and bulk solution. Error bars represent the standard deviation of three measurements of a sample. **c**–**e** Schemes of ion transport in 1D MIL-53(MP), 2D Al-TCPP, and 3D UiO-66 channels. Ions undergo multiple dehydration-rehydration processes in 3D MOF channels of window-cavity structure, resulting in low ion conductivity and mobility.

To investigate ion transport properties in MOF channels, we measured current-voltage (*I-V*) curves of MOF channels in 0.1 M chloride salts, including HCl, LiCl, NaCl, KCl, CaCl_2_, MgCl_2_, and AlCl_3_. The chloride salts current values for the MIL-53(MP) channels are nearly one order of magnitude higher than those of Al-TCPP channels and two orders of magnitude higher than those of UiO-66 channels (Supplementary Fig. 7). Combined with the drift-diffusion measurements (Supplementary Fig. 8), the cation and anion conductivity and mobility can be calculated (see “Methods” for more details). The conductivities of monovalent cations of the MIL-53(MP) channel are close to those of bulk solutions, while the divalent and trivalent cation conductivities of the MIL-53(MP) channel are lower than those of bulk solutions. The cation conductivities of the MIL-53(MP) channel are an order of magnitude higher than those of Al-TCPP channels and two orders of magnitude higher than those of UiO-66 channels (Fig. 3a). The cation mobility displays the same trend as the conductivity (Fig. 3b). As shown in Fig. 3c, the sizes of the 1D MIL-53(MP) channels around 5.2 × 8.9 Å are similar to the diameters of hydrated monovalent cations. Thus, the cations can transport through MIL-53(MP) channels without much deformation of their hydrated shells or dehydration, leading to the conductivities of monovalent cations close to those of bulk solutions. Hydrated divalent and trivalent cations with larger diameters may undergo a higher hindrance when passing through MIL-53(MP) channels, leading to lower conductivities than those of bulk solutions. For Al-TCPP channels with a 1D channel of ~8 Å inter-linked by a 2D interlayer space of ~6 Å, monovalent cations can be transported through the 1D channels without restriction. The cations appear to be partially dehydrated when they enter the 2D interlayer space from the 1D channels, showing the order of: H^+^ > M^+^ (K^+^, Na^+^, Li^+^) > M^2+^ (Ca^2+^, Mg^2+^) > Al^3+^ due to the size sieving, while the order of monovalent cations still follows K^+^ > Na^+^ > Li^+^ (Fig. 3d). For cations to be transported through 3D UiO-66 channels, they need to enter a tetrahedral cavity through a window and pass through another window to enter the octahedral cavity, following a continuous dehydration-rehydration process (Fig. 3d). Consequently, with decreasing channel size and increasing configuration complexity, the conductivities of both cations and anion decrease significantly, as do the ion mobilities. Moreover, cation transport follows the order of: H^+^ > M^+^ (K^+^, Na^+^, Li^+^) > M^2+^ (Ca^2+^, Mg^2+^) > Al^3+^ owing to the size sieving nature of the 6 Å windows, while the order of monovalent cations is reversed following the sequence of: Li^+^> Na^+^ > K^+^, due to the sieving of the window-cavity channel structure. The conductivity and mobility of the anion Cl^−^ for MIL-53(MP) channels are nearly one order of magnitude higher than for Al-TCPP channels, and two orders of magnitude higher than UiO-66 channels (Supplementary Fig. 9).

### Concentration- and pH-dependent ion transport properties

**Fig. 4:**
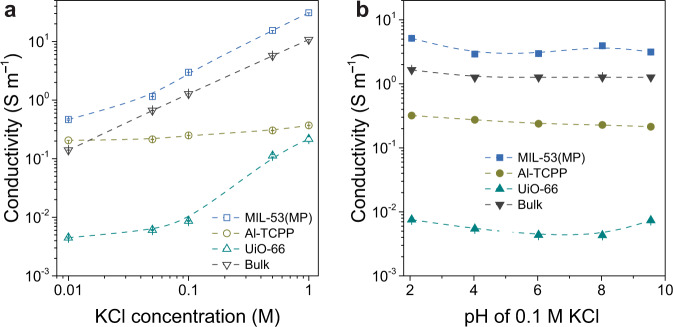
Effects of concentration and pH on the ion transport properties in MOF channels. **a** Conductivities of KCl in MOF channels measured in 0.01–1.0 M solutions. **b** Conductivities of KCl in MOF channels at 0.1 M solution with pH values from 2–10. Error bars represent the standard deviation of three measurements of a sample.

The influence of concentration on ion transport in MOF channels was investigated by measuring the *I*-*V* curves in different concentrations of KCl solution (Supplementary Fig. 10). As the KCl concentration increases from 0.01 to 1.0 M, the conductivities of MOF channels increase by one to two orders of magnitude, similar to that of the bulk solution (Fig. 4a). The pH effects on ion transport in MOF channels were also studied (Supplementary Fig. 11). For MOF channels with no functional groups, the channel surface charge is less sensitive to the changing pH. Consequently, the conductivities of the three MOF channels vary slightly (Fig. 4b). For comparison, the conductivity of the PET channel increases as pH increases from 2 to 10 (Supplementary Fig. 12), since more carboxylic acid groups get deprotonated and the channel surface becomes more negatively charged, attracting more cations passing through the channel. In addition, the conductivities of MIL-53(MP) channels are similar to those of the bulk at different KCl solution concentrations and pH.

### MD simulations

**Fig. 5:**
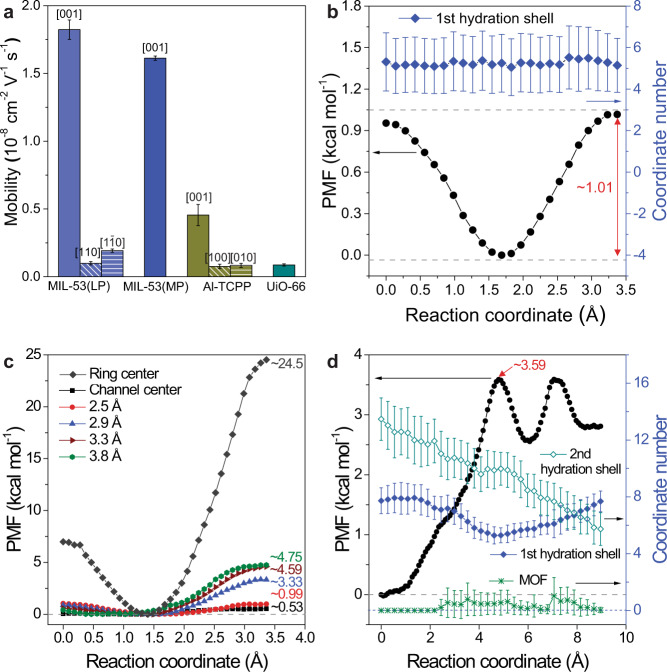
MD simulations of ion transport properties in MOF channels. **a** Electro-mobilities of K^+^ calculated from MD simulations in different MOF channels and *E*-field directions. Error bars represent the standard deviation of calculations. Note that UiO-66 channel exhibits the same ion transport property in three directions attributed to the symmetry of the cubic crystal system. Thus, the *E*-field along the [111] direction was investigated in the UiO-66 cells. **b** The PMF curve and first shell water hydration number of MIL-53(MP) channel. **c** The PMF curves of Al-TCPP channel at different channel positions. Note that the colored lines mark the profile passing through the 1D channel centre or the position of offsets from the centre and the grey line is that passing through the centre of porphyrin ring. **d** The PMF curve and the coordinate number of UiO-66 channel. The solid and open squares mark the changing of water molecules in the first and second hydration shells, respectively. The green crosses indicate the direct contact with MOF atoms (i.e., the number of MOF atoms within the first hydration layer, <~3.6 Å). All error bars represent the standard deviation of the results by the 5 sequential time intervals. Each interval lasts about 2~3 ns.

To gain insight into the experimental ordering of electro-mobility, molecular dynamics simulations (MD) were conducted to explore the electro-kinetic behaviour of ions confined within these MOF channels (Supplementary Fig. 13). Attributed to the flexibility of MIL-53, three different pore structures (i.e., large pore, MIL-53(LP); medium pore, MIL-53(MP); narrow pore, MIL-53(NP)) were investigated (Supplementary Table 1). Note that the simulated structure of MP is obtained from the density functional theory (DFT) calculation based on the XRD pattern and pore size distribution of the experimental MIL-53 with mixed LP and NP phases. The unit-cell of MIL-53 is flattened from the original LP form (16.675 × 12.813 × 6.609 Å) to the MP form (18.243 × 10.403 × 6.744 Å). Such reduction of pore size in the *y*-direction (~2.410 Å) is close to the change of pore size distribution (8.5 × 8.5 Å for MIL-53(LP) versus 5.2 × 8.9 Å for experimental MIL-53(MP) channel). Additionally, ~38.8% pore volume reduction from LP to MP was estimated by using the filled equilibrium water number in the MD simulations. The decreased pore volume calculated from MD is consistent with the pore volume reduction (~37.5%) obtained by the N_2_ adsorption-desorption measurement. The ion mobility values calculated by MD simulations follow the same order as observed in the experiments, namely MIL-53(LP) > MIL-53(MP) > Al-TCPP > UiO-66 (Fig. 5a). MIL-53(LP) shows the highest ion mobility along the 1D channel (i.e., 1.824 × 10^−8^ m^2^ V^−1^ along the [001] direction). The electro-mobilities along the directions of [110] and [$1\bar{1}0$] were also calculated. As shown in Supplementary Fig. 14, the vertically aligned benzene rings block ion transport and force ions to pass through the narrow gap between adjacent benzene rings (~4 Å). Thus, the electro-mobilities in the two directions are reduced to 0.099 and 0.192 × 10^−8^ m^2^ V^−1^, respectively. The mobility in the latter direction is approximately twice that of the former, because the [$1\bar{1}0$] direction is more favourable for mass transport, with an overlapped organic ligands alignment, while the alignment pattern is staggered in the [110] direction.

However, the electro-mobility of MIL-53 depends on its pore structure. The narrower pore size in MIL-53(MP) results in a slightly lower electro-mobility (1.612 × 10^−8^ m^2^ V^−1^) along the [001] direction and the vanishing motilities along the [110] and [$1\bar{1}0$] directions. It might be attributed to the enhanced nano-confinement effect in the MIL-53(MP) channel (Supplementary Fig. 15). With the further pore deformation, the ion transport is almost blocked in the NP form with the ignorable K^+^ mobility of 3.672 × 10^−11^ m^2^ V^−1^ s^−1^. It is due to the extremely narrow channel size (2.60 Å) where the dimension in the *y*-direction of the unit-cell (Supplementary Fig. 16) is closed to the Van der Waals diameter of the water molecule (2.82 Å)^39^. Supplementary Fig. 13d illustrates the single-file water in the MIL-53(NP) channel. For MIL-53(MP), the adsorption experiments were conducted by using MOF powders to adsorb different cations including Li^+^, Na^+^, K^+^, Ca^2+^, Mg^2+^, and Al^3+^. From the XPS results (Supplementary Table 2), the atomic concentration of Li^+^, Na^+^, K^+^, Ca^2+^, and Mg^2+^ is 0, indicating no adsorption of these cations by MIL-53(MP); while for Al^3+^, the atomic concentration (6.2%) is slightly lower than the original one (7.0%), also indicating no ion adsorption by MIL-53(MP). Similarly, the specific ion adsorption is not observed during the simulation of MIL-53(MP) channel.

The mobility inside Al-TCPP channels was studied in three directions as well. Similar to results for MIL-53, the highest mobility is reported along the 1D channel (i.e., [001]). Nevertheless, the K^+^ mobility (0.456 × 10^−8^ m^2^ V^−1^ s^−1^) is lower than that in MIL-53(MP) and bulk solution, which can be partially ascribed to the narrower channel size of Al-TCPP 1D channels. Additionally, the mobilities along the 2D interlayer spacing are further reduced to 0.076 and 0.084 × 10^−8^ m^2^ V^−1^ s^−1^ in the [100] and [010] directions, respectively. As illustrated in Supplementary Fig. 17, the parallel organic ligands form a 2D interlayer space with a width of ~6 Å. The channel width is even slightly smaller than the Van der Waals diameter of K^+^ ($2\sigma \approx$ 6.12 Å) in our MD simulations. Accordingly, the entrance of the 2D interlayer space from the 1D channel strip water molecules away from K^+^, inducing an extra energy penalty. For example, the first hydration number decreased from about 6~8 in the 1D channel (i~v) to 3~4 at the centre of the porphyrin ring (vi) (Supplementary Fig. 18). It is therefore not surprising to find reduced ion mobilities in these directions. Moreover, MD simulations results indicate that some cations could be adsorbed onto the centre of the porphyrin rings, reducing the ion mobility further (Supplementary Fig. 19). Like the previous study on 2D Al-TCPP membranes, the specific ion binding by Al-OH groups decreased the ion flux^40^. Finally, the mobility in UiO-66 channels is remarkably low with a value of ~0.087 × 10^−8^ m^2^ V^−1^ s^−1^, which can be attributed to the complex 3D window-cavity channel structure of UiO-66. For UiO-66, previous work using UiO-66 membranes for desalination demonstrated that there was no adsorption of cations by this MOF^41^. Therefore, there should be no specific adsorption between the cations and the metal centres/organic linkers of MIL-53 and UiO-66, and we chose not to discuss the adsorption effect for them. Note that the result is still consistent with our previous MOF simulation results, including Cl^–^ in UiO-66 (~0.06 × 10^−8^ m^2^ V^−1^ s^−1^)^30^ and K^+^ in UiO-66-(COOH)_2_ (~0.026 × 10^−8^ m^2^ V^−1^ s^−1^)^31^.

To explain the simulated electro-mobilities in the MOF channels, the potential of mean force (PMF) of K^+^ along a particular direction or straight pathway was investigated and appears in Fig. 5b–d, along with the changes of hydration number and MOF coordinated number. As expected, 1D MIL-53(MP) channels show a low energy barrier of ~1.01 kcal mol^−1^ at the end of the pathway (Fig. 5b and Supplementary Fig. 20a). Meanwhile, there are no significant changes in hydration shell structure or in the MOF wall contact during ion migration in MIL-53(MP) channels, indicating that the entire 1D channel of MIL-53(MP) is large enough to accommodate the hydrated K^+^ ion with a slightly lower hydration number than that in bulk solution (5.24 ± 1.38 vs. 6.71). Similarly, the PMF profile in the MIL-53(LP) form was also investigated using the similar configuration (Supplementary Fig. 20b). The lower energy barrier (0.65 kcal mol^−1^) and higher hydration number (6.81 ± 1.12) endow MIL-53(LP) with its exceptional electro-kinetic performance (Supplementary Fig. 21).

Unlike the classic harmonic constraint in MIL-53 PMF calculation, the tested K^+^ in Al-TCPP channels is also under a linear force restriction that samples the free energy profile only on a particular pathway. Fig 5c illustrates the irregular PMF profiles at different positions of the 1D Al-TCPP channel. In particular, the lowest energy barrier of ~0.53 kcal mol^−1^ is reported at the channel center, with a general removal of the hydration water molecules from 8.18 ± 1.02 to 6.95 ± 0.88 (Supplementary Fig. 22). Note that the first hydration number in the centre pathway is somewhat higher than that in bulk solution (i.e., 6.95–8.18 vs. 6.71). Nevertheless, the heterogeneous microstructure of Al-TCPP significantly shifts the energy barrier with the departure from the channel centre. Along the pathway of 2.5 Å offset (ii), the energy barrier is doubled (i.e., 1.04 kcal mol^−1^), and the average hydration number becomes lower than the bulk value without the direct MOF contact (Supplementary Fig. 22). At the maximum 3.8 Å offset (v), substantial contact with MOF atoms seriously alters the hydration shell (i.e., the hydration number is ~4) and drives the energy barrier up to 4.75 kcal mol^−1^. The PMF profile across the porphyrin ring was studied as well. The results show that the porphyrin ring with organic ligands forms an impermeable wall on the 2D interlayer spacing with the energy barrier as high as ~24.50 kcal mol^−1^. Consequently, the superiority of the lowest energy barrier of ~0.53 kcal mol^−1^ at the centre of the 1D Al-TCPP channel is compromised by the irregular PMF profile and the ion capture by porphyrin rings that deteriorate the electro-kinetic performance.

The PMF results for UiO-66 channels (Fig. 5d) were obtained under a similar linear force constraint, which represents the changes in free energy along the pathway connecting the centers of MOF cavities and crossing the windows. The dehydration-rehydration process at the windows, in conjunction with the insignificant MOF contact, elevates the energy barrier up to i.e., ~3.59 kcal mol^−1^, giving the lowest ion mobility. As in our previous study^31^, the rehydration of the first hydration shell only moderately recovers the free energy. Due to tetrahedral cavity confinement, the extension of second hydration shell is diminished, giving rise to the extra energy penalty^30^ (open squares in Fig. 5d). Note that the resultant energy barrier is lower than from previous studies of Cl^–^ in UiO-66 (~7.00 kcal mol^−1^) and K^+^ in UiO-66-(COOH)_2_ (~5.56 kcal mol^–1^)^31^, owing to the employed generic MOF force-fields.

**Fig. 6:**
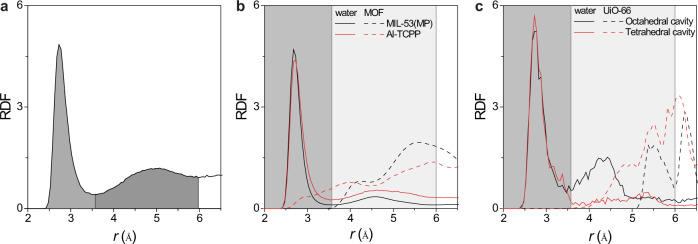
Radial distribution function (RDF) profiles of K+ ion in bulk solution and MOF channels. **a** The RDF profile for K^+^-(OH_2_) in 1.0 M KCl solution. The grey and dark grey regions mark the first and second hydration shell structures. **b**, **c** The RDF of K^+^ in MIL-53(MP), Al-TCPP, and UiO-66 channels. Note that the solid and dashed curves represent the RDF with water molecules and MOF atoms, respectively. The dark and grey regions in (**b**) and (**c**) are the extension of the first and second hydration shells, respectively.

To gain further molecular level insights into ion mobilities in MOF channels, the radial distribution functions (RDF) of the cation to the water molecule (i.e., oxygen atom) and solid MOF atoms were plotted in Fig. 6. In the bulk solution (Fig. 6a), there are two distinct hydration shells for the K^+^ ion with radii of ~3.6 and ~6.0 Å, respectively. The water RDFs of MIL-53(MP) and Al-TCCP channels are compared in Fig. 6b, where the results are a statistical average of all free cations in the thermal equilibrium stage (5 ns). Although the water RDF functions indicate a similar first hydration shell structure in the two MOF channels with values of 6.14 (MIL-53(MP)) and 5.49 (Al-TCCP), there are distinct differences in the MOF profiles. The emergence of MOF atoms in the range of 2.7–3.5 Å in Al-TCPP suggests the capture of ions by Al-TCPP to a certain degree, especially for ions confined in the 2D interlayer and specifically those bound to the porphyrin rings. The ion capture effect, together with the heterogeneous network, i.e., 2D interlayer space vertically inter-connected 1D channels, plays an essential role in ion transport properties. Compared with Al-TCPP, all the ions in MIL-53(MP) channels are confined within the same environment (e.g., ~5.2 Å × 8.9 Å 1D channel). Such a straightforward 1D channel composed of aligned benzene rings can facilitate electro-kinetic ion transport in the [001] direction, resulting in the highest ion mobility. The hydration structure of the K^+^ ion is significantly dependent on its position inside UiO-66 channels. Fig. 6c illustrates the RDF of K^+^ fixed on the centre of tetrahedral and octahedral cages. Obviously, the first hydration shell remains intact in both positions (7.65 vs. 7.80), while the hydration number in the centre of the tetrahedral cage drops considerably reducing from 19.81 to 12.58. The organic ligands from *r* $\approx$ 4.5 Å prevent the formation of a second hydration shell. Thus, ion migration inside UiO-66 channels inevitably encounters a continuous energy barrier for partial dehydration of the second hydration shell, giving rise to the lowest ion mobility.

Although the ion mobilities calculated by MD simulation are slightly lower than those observed in experiments, MD reproduced the same sequence of mobilities for K^+^ in the three MOF channels in the same order of magnitudes. The discrepancy may be attributed to a combination of the generic MOF force-field covering most MOFs and the empirical atomic charge prediction methods^42,43^. The resultant mobility is closely related to the channel configuration, channel interconnectivity, and the specific binding of ions by functional sites. Therefore, MIL-53(MP), with unique straightforward 1D channels and binding-free surface chemistry, is a potential material for optimizing ion transport in angstrom-scale channels. On the other hand, ion captures, along with the heterogeneous network in Al-TCPP (i.e., 1D channel vertically interconnected by 2D interlayer spacing), reduces the ion transport capability.

## Discussion

Angstrom-scale ion channels with versatile pore configurations and sizes have been successfully constructed to investigate angstrom-confined ion transport. The experimental and theoretical simulation results reveal that the pore structures of MOFs significantly influence ion transport rates and sieving properties under conditions of angstrom-scale confinement. Ion mobilities decrease by orders of magnitude as the channel configurations vary from 1D to 3D. The 1D configuration with flexible pore size around 5.2 Å × 8.9 Å can facilitate cations transport in MIL-53 channels. In contrast, MOF channels with hybrid pore configurations and sizes, such as Al-TCPP channels of ~8 Å 1D channels connected by 2D ~6 Å interlayers and 3D UiO-66 channels of ~6 Å windows and 9 − 12 Å cavities, impose higher energy barriers for ion transport due to the multiple dehydration-rehydration effects. This work opens ways to fabricate artificial sub-1-nm ion channels based on angstrom-porous MOFs with a variety of channel configurations as building blocks, offering potential applications in highly efficient ion separation and energy conversion technologies.

## Methods

### Materials

Aluminum nitrate nonahydrate (Al(NO_3_)_3_^.^9H_2_O), zirconium chloride (ZrCl_4_), terephthalic acid (BDC), dimethylformamide (DMF), sodium dodecyl diphenyloxide disulfonate, formic acid (HCOOH), and hydrochloric acid (HCl) were purchased from Sigma-Aldrich, Australia. TCPP (5,10,15,20-tetrakis(4-carboxyphenyl) porphyrin) was obtained from TCI. Methanol, ethanol, potassium hydroxide (KOH) and sodium hydroxide (NaOH) were purchased from Merck. PET membranes were ordered from Hostaphan RN12 Hoechst. Milli-Q water was used throughout the experiments.

### Powder X-ray diffraction (PXRD)

PXRD patterns were recorded in the 2θ range of 2–50° at room temperature using a Miniflex 600 diffractometer (Rigaku, Japan) in transmission geometry using Cu Kα radiation (15 mA and 40 kV) at a scan rate of 2° min^−1^ and a step size of 0.02°. These synthesized MOFs were washed with DMF for three times every other day, and with methanol following the same procedure, and then followed by drying at 80 °C for 12 h in the oven. The as-prepared MOF powders were stored in a capped glass vial and used for the XRD characterization.

### Scanning electron microscopy (SEM)

SEM images were taken with a field-emission scanning electron microscope (FEI Magellan 400 FEG SEM) operating at 5 kV, 13 pA.

### Gas adsorption and desorption measurement

For gas adsorption/desorption isotherms, high-purity grade (99.999%) nitrogen was used in all measurements. Prior to measuring the gas adsorption/desorption, MIL-53 powders were degassed at 80 °C (or 150 °C) for 24 h, while Al-TCPP and UiO-66 powders were degassed at 150 °C for 24 h. Low pressure volumetric nitrogen adsorption isotherms up to 1 bar were measured using a micromeritics 3 Flex gas sorption analyzer. Pore size distributions were determined by measuring N_2_ adsorption/desorption isotherms at 77 K in a liquid nitrogen bath and calculated using the Micromeritics software with Horvath-Kawazoe (H-K) model for MIL-53, and N_2_-Density Functional Theory (DFT) model for Al-TCPP and UiO-66.

### Nanochannel preparation

Single PET nanochannel membranes (12 μm thick) were simultaneously etched from one side with 6.0 M NaOH+ 0.025% sodium dodecyl diphenyloxide disulfonate and from the other side with 6.0 M NaOH etching solution at 60 °C to produce single bullet-like nanochannels. A Keithley 6487 picoammeter (Keithley Instruments, Cleveland) was employed to observe the changing current of the single-nanochannel membrane during etching. The etching process was terminated by adding a mixture of 1.0 M KCl-HCOOH aqueous solution, which neutralized the alkaline etching solution. The morphologies and diameters of the nanochannels were observed by SEM using multichannel membranes prepared under the same etching conditions as single channel membranes.

### MOFs channel preparation

Three MOFs—MIL-53, Al-TCPP, and UiO-66—were prepared by solvent-thermal synthesis at 120 °C for 24 h. For the synthesis of MIL-53 seeds, 300 mg Al(NO_3_)_3_^.^9H_2_O and 300 mg BDC were dissolved in 25 mL DMF in a vial; for Al-TCPP seeds, 375 mg Al(NO_3_)_3_^.^9H_2_O and 395 mg TCPP were dissolved in 25 mL DMF in a vial; for UiO-66 seeds, 466 mg ZrCl_4_ and 332 mg BDC were dissolved in 25 mL DMF in a vial. The obtained MOFs were washed with DMF and methanol three times, and then were used as seeds for the MOF channel fabrication. A single-nanochannel PET membrane was clamped between the two cells, which were filled with the MOF seed aqueous solution with a concentration of 0.1 mg mL^−1^. The MOF seeds were driven into the PET channel at 1 V for 2 h.

Al(NO_3_)_3_^.^9H_2_O (300 mg) and BDC (300 mg) in DMF (4 mL) were dissolved in two vials separately at 90 °C. The obtained clear solution was transferred into an interfacial reaction holder in a Teflon-lined autoclave with the seeded single-nanochannel PET membrane placed vertically between the two cells, the metal solution in the large base side and ligand solution in the small base side. The autoclave was then transferred to an oven and heated at 120 °C for 24 h. After cooling to room temperature, the as-prepared MIL-53 channel was washed with methanol three times, followed by drying at 80 °C overnight. As for Al-TCPP channels, Al(NO_3_)_3_^.^9H_2_O (37.5 mg) and TCPP (39.5 mg) in DMF (4 mL) were dissolved in a vial at 90 °C, and for UiO-66 channels, ZrCl_4_ (46.6 mg) and BDC (33.2 mg) in DMF (4 mL) were dissolved in a vial at 90 °C. The subsequent procedures are similar to those used in the preparation of MIL-53 channels.

### Current measurement

Current measurements were carried out with a Keithley 6517B picoammeter (Keithley Instruments, Cleveland), and the MOF or PET nanochannel membrane was placed between two cells. The Ag/AgCl electrode was inserted into each cell and employed to apply a voltage across the nanochannel. The small base side of the nanochannel faced the anode, and the large base side faced the cathode. A scanning voltage from −2 V to +2 V with a period of 20 s was scanned three times. To measure the ion transport properties of the nanochannel, different salt solutions were added to both cells. KCl solutions with various concentrations (0.01 M, 0.05 M, 0.1 M, 0.5 M, and 1.0 M) and pH values (2–10) were also measured. Then the ionic currents during the potential scan were recorded.

### Ion conductivity and mobility in MOF channels

The ionic conductivity (*k*) of a nanochannel can be defined as:

1 $$\kappa=G\cdot \frac{L}{S}$$

where *G* is the conductance of a nanochannel, *S* is its cross-sectional area, and *L* is its length. For MOF channels, *S* is the effective cross-sectional area of MOF pores.

For a bullet-shaped nanochannel, the radius profile *r(x)* can be described as:

2 $$r(x)=\frac{{r}_{b}-{r}_{t} \exp \left(-\frac{L}{h}\right)-({r}_{b}-{r}_{t}) \exp \left(-\frac{x}{h}\right)}{1- \exp \left(-\frac{L}{h}\right)}$$

where *r*_*b*_ is the base radius, *r*_*t*_ is the tip radius, *L* is the length of a nanochannel, and *h* is a geometrical parameter characterizing the curvature of a nanochannel profile, designated the curvature radius, which was obtained by fitting the obtained experimental tip profiles^44,45^. The average *L*/*S* value of a bullet-shaped nanochannel is theoretically described as:

3 $$\frac{L}{S}=\frac{{L}^{2}}{{\int }_{0}^{L}\pi {r(x)}^{2}{dx}}=\frac{{L}^{2}}{{\int }_{0}^{L}\pi {\left(\frac{{r}_{b}-{r}_{t} \exp \left(-\frac{L}{h}\right)-({r}_{b}-{r}_{t}) \exp \left(-\frac{x}{h}\right)}{1- \exp \left(-\frac{L}{h}\right)}\right)}^{2}{dx}}$$

At high electrolyte concentration (i.e., 1.0 M) and a pH value close to the isoelectric point of 3.8 at the surface, where the electrical double layer can be neglected and the specific ion conductivity in a nanochannel is equal to that in bulk solution, the (*L*/*S*)_NC_ of a nanochannel can be calculated by:

4 $${\left(\frac{L}{S}\right)}_{{NC}}=\kappa \frac{U}{I}$$

where *k* is the ion conductivity of 1.0 M electrolyte in bulk solution, and *I* is the ion current measured at the applied voltage *U*. For MOF channels, (*L*/*S*)_MOF_ is calculated by:

5 $${\left(\frac{L}{S}\right)}_{{MOF}}={\left(\frac{L}{S}\right)}_{{NC}}.\frac{1}{{\nu }_{{MOF}}{d}_{{MOF}}}$$

where *v* is the pore volume of MOF crystals and *d*_calc_ is the calculated crystal density (Supplementary Table 3).

Drift-diffusion experiments were performed with voltages from −0.1 V to +0.1 V applied via Ag/AgCl electrode. The PET or MOF channel membrane was placed between two cells, one facing the tip side, filled with 0.01 M chloride salt solution, and the other facing the base side, filled with the same chloride salt solution at a concentration of 0.1 M. The concentration gradient of electrolytes (*Δ*) across the membrane is 10. From the resulting *I-V* curves (Supplementary Fig. 8), the measured reverse potential (*E*_*m*_) was obtained. During these measurements, a redox potential was generated at the electrodes because of the concentration gradient across membrane; thus, *E*_*m*_ is subtracted from the redox potential *E*_redox_, which is theoretically calculated as follows:

6 $${E}_{{redox}}=\frac{{RT}}{{zF}}{{{{{\rm{ln}}}}}}\triangle \frac{{\gamma }_{{C}_{H}}}{{\gamma }_{{C}_{L}}}$$

where *R*, *T*, *z*, and *F* are the universal gas constant, temperature, ion valence, and the Faraday constant, respectively. The *γ* with subscript of *H* and *L* stand for the activity coefficient of high (0.1 M) and low (0.01 M) concentration, respectively. Thereafter, the real reverse potential *E*_*R*_ was calculated by^20^:

7 $${E}_{R}={E}_{m}-{E}_{{redox}}$$

Based on *E*_*R*_, the mobility ratio of cation (${\mu }_{+}$) against anion (${\mu }_{-}$) can be obtained following the Henderson equation^46^ with the valence of the cation (${z}_{+}$) and anion (${z}_{-}$) in consideration:

8 $$\frac{{\mu }_{+}}{{\mu }_{-}}=-\left(\frac{{z}_{+}}{{z}_{-}}\right)\left(\frac{{{{{{\rm{ln}}}}}}\triangle -{z}_{-}F{E}_{R}/{RT}}{{{{{{\rm{ln}}}}}}\triangle -{z}_{+}F{E}_{R}/{RT}}\right)$$

The chloride salt conductivities can also be calculated by

9 $$\kappa=F\times \left({c}_{+}{\mu }_{+}+{c}_{-}{\mu }_{-}\right)$$

${c}_{+}$and ${c}_{-}$ are the concentration of cation and anion, respectively. Thus, the cation mobility (${\mu }_{+}$) and anion mobility (${\mu }_{-}$), and cation conductivity (${\kappa }_{+}$) and anion conductivity (${\kappa }_{-}$) can be calculated.

### DFT calculation

To more accurately identify the crystal structures of the experimental MIL-53 channel, we conducted the density functional theory (DFT) calculation using Vienna Ab-initio Simulation Package (VASP)^47^. The initial structure of DFT calculation is the same as the well-established MIL-53(LP). Then it was except for compressed along the y-dimension, i.e., from 16.675 × 12.813 × 6.609 Å to 16.675 × 10.038 × 6.609 Å. After that, the geometry and the unit-cell of MIL-53 were fully optimized until the forces were smaller than that of 0.02 eV Å^−1^. During the DFT calculation, the cut-off of plane-wave was set to be 500 eV and the exchange-correlation functionality is approximated by the Perdew-Burke-Ernzerhof (PBE)^48^ functionality. The projector augmented wave method (PAW)^49^ was employed to represent the core electron. The optimized unit-cell is 18.243 × 10.403 × 6.744 Å, which is named as the medium pore structure, MIL-53(MP). The pore structure of the obtained MIL-53(MP) is largely consistent with the experimental XRD pattern and N_2_ adsorption-desorption measurement.

### MD simulation

To investigate the electro-kinetic behavior of ions in different MOF channels, we conducted a series of non-equilibrium molecular dynamics simulations (NEMD) of the electro-kinetic behavior of ions confined within the interior of MOF channels. In the experimental study, the overall electro-mobilities showed a sequence of MIL-53(LP) > MIL-53(MP) > Al-TCPP > UiO-66> MIL-53(NP). Therefore, our MD simulation focused on explaining this phenomenon and the electro-kinetic transport of 1.0 M KCl solution was theoretically explored as an example. Supplementary Fig. 13 illustrates six different simulation cells including bulk solution, MIL-53(LP), MIL-53(MP), MIL-53(NP), Al-TCPP, and UiO-66 channels. Attributed to the intrinsic anisotropic microstructure, the mobilities of MIL-53 and Al-TCPP were investigated in three different directions (one along 1D channels of MIL-53 and Al-TCPP and another two normal directions, e.g. along the 2D interlayer spacing in Al-TCPP).

The number of water molecules in each MOF cell was determined from a trial-and-error process where the cell was filled with different numbers of water molecules and tested until the pressure of water molecules approach ambient conditions (~1 atm). Each test case was equilibrated at 300 K for 1.5 ns and the average pressure in the last 1 ns was recorded. The equilibrium bulk solution was determined via a similar trial-and-error process of the dimension of simulation cells rather than the number of water molecules. Then, different numbers of cation-anion pairs were randomly added into the simulation cells to approach the same concentration as that in the experiment reservoir (i.e., 1.0 M). The details of each MD simulation configurations were summarized in Supplementary Table 4, where the concentration of the electrolyte was estimated from the water-ion ratio.

Once the equilibrium configuration was determined, each MD simulation cell was further equilibrated at 300 K for another 7 ns after a thermal annealing process from 1800 K to 300 K. The trajectory in the last 5 ns was recorded for the post-analysis (i.e., RDF). After 7 ns equilibrium, NEMD was conducted by imposing an external electric field (*E*-field). Using an external *E*-field to calculate ion mobility has been widely adopted in MD simulations for investigating the ion transport in the nano-channels^50–53^. The employed *E*-field strength (i.e., 0.1 V Å^−1^ ^31,50,54^) has been used frequently, which is still in the linear response regime^50,54^ and is still less than the upper *E*-field limitation (1.0 V Å^−1^), where the polarization of water molecules can be neglected^55,56^. The effects of the *E*-field strength on the K^+^ mobility in the MIL-53(MP) framework were investigated. The calculated mobility is marginally decreased with the strength of the *E*-field (Supplementary Fig. 23). Each NEMD simulation continued for 15~20 ns, and the last 10~15 ns were taken for the post-analysis. The long simulation duration is designed to increase the statistical convergence.

Thus, the electro-mobility of ions is derived from

10 $${\bar{\mu }}^{\pm }=\pm \frac{1}{\triangle {tN}}{\sum }_{\alpha }^{N}\frac{{S}_{\alpha }^{\pm }.{n}_{E}}{{{{{{\rm{|}}}}}}E{{{{{\rm{|}}}}}}}$$

where ${{{{{{\rm{s}}}}}}}_{\alpha }^{\pm }$ is the displacement vector in three dimensions for $\alpha$th cation or anion, during a period of time, $\varDelta t$. ${{{{{{\rm{n}}}}}}}_{{{{{{\rm{E}}}}}}}$ is the unit vector, pointing in the direction of the *E*-field. $\left|E\right|$ is the strength of the *E*-field as 0.1 V/Å. $N$ is the total number of cations or anions in a simulation cell. Owing to the intrinsic anisotropy microstructure of MIL-53 and Al-TCPP, the *E*-fields along three different directions have been studied in these two MOFs, as illustrated in Supplementary Figs. 14–17. Taking Al-TCPP for example, the *E*-field along [001] direction mimics the ion migration along the 1D channel, while the *E*-field along another the other two directions (*i.e*., [100] and [010]) mimics the ion transport inside the 2D Å interlayer. Due to the symmetry of the cubic crystal system, the *E*-field along only the [111] direction or the *x*-axis was investigated in the UiO-66 cells and the bulk solution.

To simulate the three different MOF structures in MD simulations, a general MOF force-field was employed to describe its properties including the 12-6 Lennard-Jones (LJ) parameters, topology connectivity and the atomic partial charges^57^. UFF was adopted to describe the non-bonded intra- and inter-molecular interaction^58^. All the intra-molecular constraints including the bond, angle, dihedral, and improper configurations were automatically derived from the crystal structure. Finally, the atomic partial charge was derived using an empirical charge-equilibration (Qeq) method^42,43^. To confirm the validity of the obtained results, other methods such as Density Derived Electrostatic and Chemical (DDEC6) and Zero-charges were also studied (see details in Supplementary Fig. 24–25). Only Qeq methods show the consistent tendency as the experiment observation. In addition, TIP3P water was taken as the solvent molecule^59^ and was rigidified by the shake algorithm^60^. The LJ parameters of ions were taken from a highly optimized monovalent ion force-field^61^. The simulation was conducted using the LAMMPS MD package^62^. The Nosé–Hoover thermostat was used in most of the equilibrium stages^63,64^.

The main differences between the three charge allocation methods are the charge polarization of the porphyrin ring in Al-TCPP (Supplementary Fig. 25). The Qeq, DDEC, and zero-charge methods generated high, medium, and no polarization, which lead to strong, intermediate, and weak K^+^ adsorption at porphyrin rings, respectively. But the resultant K^+^ ion mobility for the intermediate and weak K^+^ adsorption cases in Al-TCPP is much higher than those in the MIL-53, which is opposite to the experiments. Therefore, the comparison clearly indicates the K^+^ strong adsorption in Al-TCPP is the key mechanism. It is widely believed that the DDEC6 is more accurate than the Qeq method. It is thus intriguing why the Qeq method leads to a trend agreement with experiments (Supplementary Fig. 24). Be aware that the aqueous ion adsorption in nanochannels is a delicate competition among ion-porphyrin affiliation, ion dehydration, and the nanoconfinement contribution. It is possible that the adopted UFF force model parameters could not accurately describe the non-electrostatic interactions in the systems so that only a high charge polarization of porphyrin (using the qeq method) in our models could correctly reproduce the K^+^ strong adsorption.

### PMF calculation

To explain the simulated electro-mobilities, we further took a series of MD simulations to reveal the potential of mean force (PMF) of ions migrating along the specific pathway in MOF channels. We investigated the K^+^ PMF curve using the collective variable module in LAMMPS^65^. Similar to our previous work^31^, we added K^+^-Cl^–^ pair in each simulation cell, where K^+^ was constrained by a harmonic spring with a strength of 40 kcal mol^–1^ Å^–2^ strength and Cl^–^ was frozen during the simulation period. Furthermore, to accelerate the calculation, the MOF supercells (*i.e*., the sizes of the simulation cells) were reduced to 1 × 2 × 10, 2 × 3 × 10, and 2 × 2 × 2 for MIL-53, Al-TCPP, and UiO-66, respectively. Each MOF study included tens of umbrella sampling windows and each sampling window lasted for 10 ns at 300 K, controlled via a Nosé–Hoover thermostat^63,64^. The trajectory of K^+^ was recorded every 1 ps and analysed via a weighted histogram analysis method, WHAM code^66^. The uncertainty of PMF results was investigated by Monte Carlo (MC) bootstrap error analysis using 10,000 MC steps (Supplementary Table 5). The statistic uncertainty for all curves is nearly more minor than that of the symbols in Fig. 5. Note that investigations were done along the direction of maximum K^+^ mobility in each MOF channel (i.e., [001] in Al-TCPP and MIL-53, and [111] in UiO-66). To clarify our PMF results, the first and second hydration and MOF coordinate numbers of K^+^ with cut-offs at *r* < ~3.6 Å and *r* < ~6.0 Å were recorded and investigated in each sampling windows (e.g. Supplementary Fig. 22 for Al-TCPP). Additionally, we compared the PMF results in the MIL-53(MP) framework with different duration times (5 ns vs 10 ns) for umbrella sampling, which show the same energy barrier of 1.01 kcal/mol (Supplementary Fig. 26). Thus, the used duration time (10 ns) in this work is long enough to produce convergent results.

Because the various MOF channels have highly heterogeneous and diverse microstructure, we used different kinds of umbrella sampling and simulation constraints in PMF calculations. First, in MIL-53(MP), there are 25 sampling windows along [001] (Supplementary Fig. 27). Spanning a half-length of unit-cell in the *z*-direction (i.e., 3.37 Å), with an inter-window spacing of ~0.14 Å. Such a configuration benefits from the symmetry of crystal structures (see in Supplementary Fig. 20). During the simulation, the testing K^+^ is free to migrate on the plane perpendicular to [001] pathway. Hence, our PMF results represent the energy barrier of the entire 1D channel in MIL-53(MP) rather than the variation of free energy on a particular pathway in the following Al-TCPP and UiO-66 studies.

Second, there are 13 sampling windows in Al-TCPP along the [001] direction that span a similar half-length of unit-cell (i.e., 3.30 Å), with a spacing of ~0.28 Å (Supplementary Fig. 28). Nevertheless, we restricted the migration of the testing K^+^ by removing the perpendicular force components in *x* and *y* directions. Hence, the results from the Al-TCPP study were essentially different from MIL-53(MP) and represent only the energy barrier along a particular pathway. Supplementary Fig. 22 illustrates the positions of pathways as (i) the channel centre, (ii) 2.5 Å offset from centre, (iii) 2.9 Å offset, (iv) 3.3 Å offset, (v) 3.8 Å offset and (vi) the centre of the porphyrin rings. The substantial changes in PMF curves at the different positions are attributed to the highly heterogeneous microstructure of Al-TCPP (Fig. 5c).

Finally, our UiO-66 studies calculated the PMF curve along [111] direction with a similar linear force constraint in the previous studies. Because the PMF pathway connects the centres of the octahedral to those of the tetrahedral cavities and crosses the centre of the MOF windows. We investigated the pathway of the minimal energy barrier. There is a total of 34 sampling windows with a spacing of 0.27 Å and a total length of ~8.96 Å, as in our previous UiO-66 studies^30,31^ (Supplementary Fig. 29).

### RDF calculation

The radial distribution functions (RDF) of K^+^ to the water molecules (i.e., oxygen atoms) in bulk water are plotted in Fig. 5, which shows distinctive hydration shell structure with first and second hydration numbers of 6.71 and 22.1 and radii of ~3.6 and ~6.0 Å, respectively. Fig 5c gives the RDF results for K^+^ in the center of the octahedral and tetrahedral cavities in UiO-66. The confinement of the tetrahedral cavity considerably stripped the second hydration shell and enhanced the energy barrier. The RDF in UiO-66 was calculated via the same methods as our previous work^31^, in which a single cation-anion pair is fixed on the center of the octahedron and tetrahedron cages. It was thermally equilibrated at 300 K for 7 ns and the trajectory in the last 5 ns was taken for post-analysis.

## Supplementary information

Supplementary Information

## Data Availability

The data that support the findings of this study are available from the paper and its Supplementary Information. Raw data are available from corresponding authors upon request.
