# Supplementary material for: Construction of angstrom-scale ion channels with versatile pore configurations and sizes by metal-organic frameworks
Source: Nat Commun. 2023 Jan 18;14:286. doi: 10.1038/s41467-023-35970-x (PMC9849445; doi:10.1038/s41467-023-35970-x)
Supplement: Supplementary file 1 — Supplementary Information [file 41467_2023_35970_MOESM1_ESM.pdf]

Supplementary Information

Construction of angstrom-scale ion channels with versatile pore configurations and sizes by metal-organic frameworks

Li et al.

Construction of angstrom-scale ion channels with versatile pore configurations and sizes by metal-organic frameworks

Xingya Li[1], Gengping Jiang[2]*, Meipeng Jian[1], Chen Zhao[3], Jue Hou[3], Aaron W. Thornton[4], Xinyi Zhang[5], Jefferson Zhe Liu[6], Benny D. Freeman[1,7], Huanting Wang[1], Lei Jiang[1], Huacheng Zhang[3]*

Affiliations:

[1]Department of Chemical Engineering, Monash University, Clayton, Victoria 3800, Australia

[2]College of Science, Wuhan University of Science and Technology, Wuhan 430072, China

[3]Chemical and Environmental Engineering, School of Engineering, RMIT University, Melbourne, Victoria 3000, Australia

[4]Manufacturing, CSIRO, Clayton 3168, Australia

[5]Hubei Key Laboratory of Ferro- & Piezoelectric Materials and Devices, Faculty of Physics & Electronic Science, Hubei University, Wuhan 430062, China

[6]Department of Mechanical Engineering, The University of Melbourne, Parkville, VIC 3010, Australia

[7]Department of Chemical Engineering, The University of Texas at Austin, Austin, TX 78712, USA.

*Correspondence: huacheng.zhang@rmit.edu.au, gengpingjiang@wust.edu.cn

Supplementary Notes:

The MIL-53 framework is flexible and thus can have multiple types of pore configurations, including large pore (LP), narrow pore (NP), and hybrid LP and NP forms under different experimental conditions (Supplementary Table 1). We have carefully analyzed experimental characterization to gather detailed quantitative information, as briefly summarized below.

(1) The as-synthesized MIL-53(Al) should be activated so that the large pore (LP) form can be generated. The studied MIL-53(Al) is synthesized using DMF as the solvent at 120 ^{o}C. After activation at 150 ^{o}C, the MIL-53(activated) is present in the large pore (LP) form evidenced by XRD and N_2 adsorption-desorption characterization. The obtained XRD pattern of MIL-53(LP) (Supplementary Fig. 5a) is similar to the simulated LP form, and the pore size distribution shows the LP form with a pore size of 8.5 × 8.5 Å (Supplementary Fig. 6b).

(2) The studied MIL-53 for ion transport experiments could have mixed large and narrow pores. The as-synthesized MIL-53 was washed with DMF to remove the residual ligands in MOF pores, then washed with methanol to exchange DMF and followed by drying at 80 ^{o}C to remove methanol, forming the MIL-53(non-activated). We immersed the MIL-53(non-activated) in an aqueous salt solution (0.1 M KCl, for example) for 12 h to prepare the MIL-53(KCl). Supplementary Fig. 5a shows the XRD patterns of MIL-53 under different conditions. The MIL-53(non-activated) and MIL-53(KCl) show very similar features that are notably different from MIL-53(LP), indicating some structural changes that are different from the pure LP form. A careful comparison with the simulated XRD pattern of the MIL-53(NP) form suggests that MIL-53(non-activated) and MIL-53(KCl) are not in the pure NP form either. Some signatures of LP and NP forms can be observed in MIL-53(KCl). In addition, N_2 adsorption-desorption measurement shows the pore volume of MIL-53(non-activated) as 0.35 $cm^3\ g^{-1}$, which is between the theoretical pore volume of LP (0.56 $cm^3\ g^{-1}$) and NP (0.27 $cm^3\ g^{-1}$) (Supplementary Table 1).

(3) A hypothetical structure termed medium pore (MP) describes the studied MIL-53 for ion transport experiments of hybrid LP and NP phases. To get more pore structure information, we also did N_2 adsorption-desorption experiments for MIL-53(non-activated). The pore size distribution results show a broad pore width from 5.2 Å to 8.9 Å (**Fig. 2a**). Note that the LP has a pore of 8.5 × 8.5 Å and the NP has a pore of 2.6 × 13.6 Å. The peak at 8.9 Å should be assigned to LP. It is rather difficult to assign the peak at 5.2 Å. It might arise from averaging the elongated pore geometry of NP. It could also be due to some medium pores (i.e., MP) between LP and NP. To construct the hypothetical MP structure for the study of ion transport, we did DFT simulations to identify the potential MP structure by purposely

shrinking one cross-section dimension of the LP framework (while other dimensions were allowed to relax). The dimension of the unit-cell was flattened from LP (16.675 × 12.813 × 6.609 Å) to MP (18.243 × 10.403 × 6.744 Å). The selected dimension reduction in the *y*-direction (~2.410 Å) is close to the pore size distribution change, from 8.5 Å to 5.2 Å, observed in the N_2 adsorption-desorption measurement. Supplementary Fig. 5a compares the simulated XRD pattern of the MP form with experimental results (MIL-53(KCl)). The peaks at 9.78^o, 15.64^o and 22.12^o agree with those of the MIL-53(KCl) very well. In addition, we estimated ~38.8% volume reduction of MP from LP by using the filled equilibrium water number in MD simulations, which is consistent with the pore volume reduction in the N_2 adsorption-desorption measurement (~37.5%).

In conclusion, the simulated MIL-53(MP) structure is constructed based on the DFT calculations in combination with the XRD pattern and pore size distribution of the experimental MIL-53 with mixed LP and NP phases.

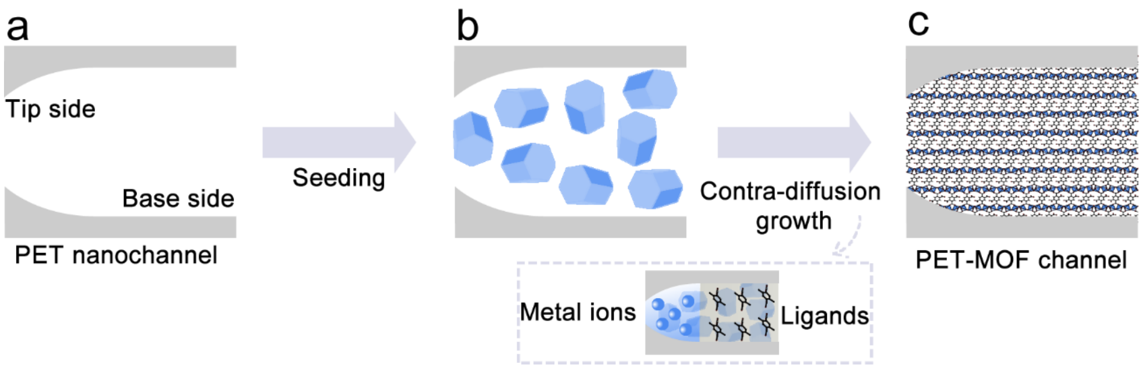

Supplementary Fig. 1. Schematic of the synthesis procedure of MOF channels. **a** Bullet-shaped single PET nanochannel. **b** MOF crystal-seeded PET nanochannel. **c** PET-MOF channel that the PET nanochannel fully filled with inter-grown MOF cystals.

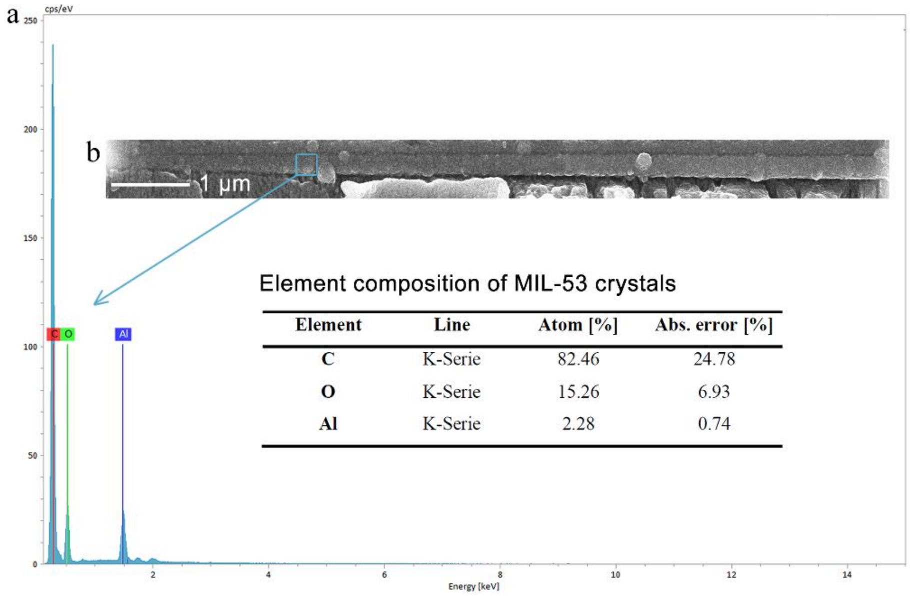

Element	Line	Atom [%]	Abs. error [%]
**C**	K-Serie	82.46	24.78
**O**	K-Serie	15.26	6.93
**Al**	K-Serie	2.28	0.74

Supplementary Fig. 2. Characterization of MIL-53(MP) channel. **a** EDX spectra and element composition of a MIL-53(MP) channel. **b** SEM images of a MIL-53(MP) channel. The “absolute error” represents the standard deviation of three measurements of a sample.

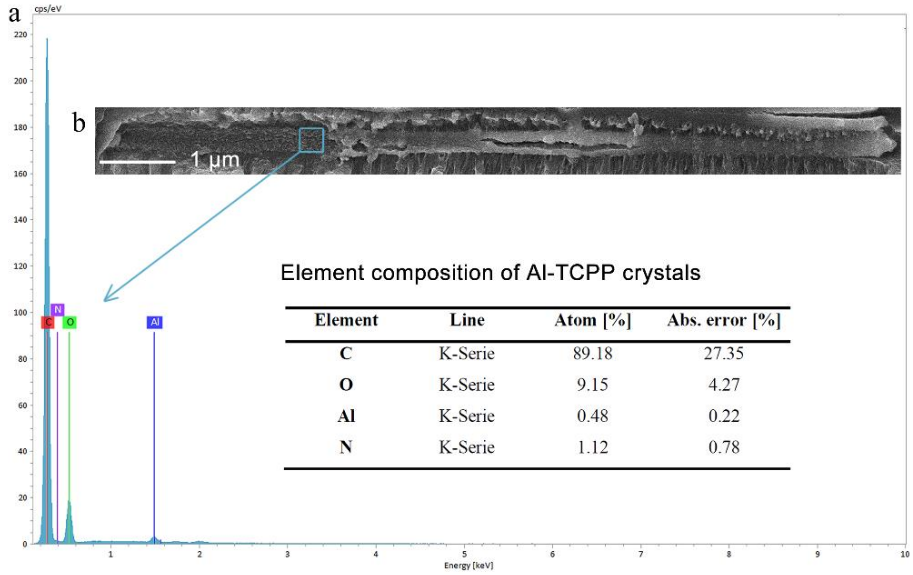

Element	Line	Atom [%]	Abs. error [%]
**C**	K-Serie	89.18	27.35
**O**	K-Serie	9.15	4.27
**Al**	K-Serie	0.48	0.22
**N**	K-Serie	1.12	0.78

Supplementary Fig. 3. Characterization of Al-TCPP channel. **a** EDX spectra and element composition of an Al-TCPP channel. **b** SEM images of an Al-TCPP channel. The “absolute error” represents the standard deviation of three measurements of a sample.

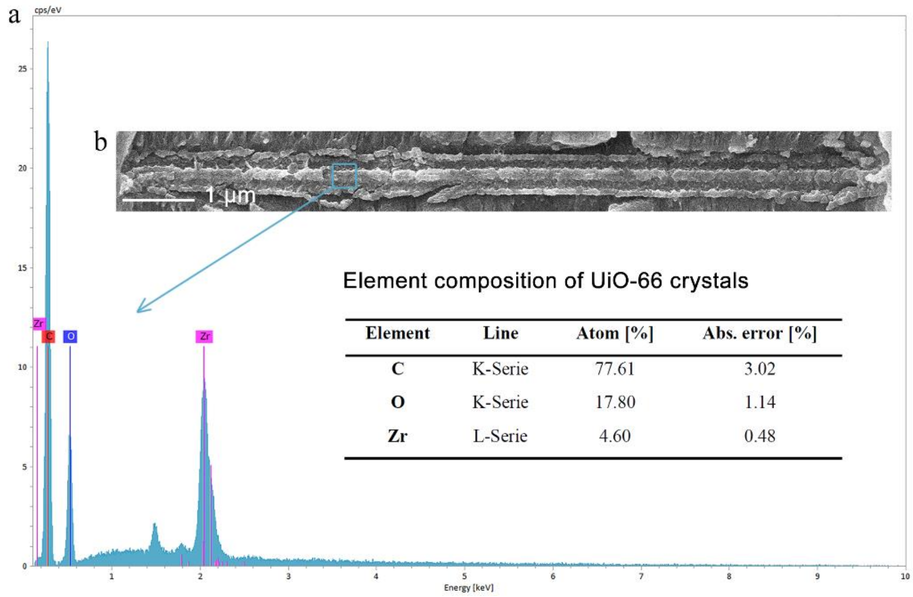

Element	Line	Atom [%]	Abs. error [%]
**C**	K-Serie	77.61	3.02
**O**	K-Serie	17.80	1.14
**Zr**	L-Serie	4.60	0.48

Supplementary Fig. 4 Characterization of UiO-66 channel. **a** EDX spectra and element composition of a UiO-66 channel. **b** SEM images of a UiO-66 channel. The “absolute error” represents the standard deviation of three measurements of a sample.

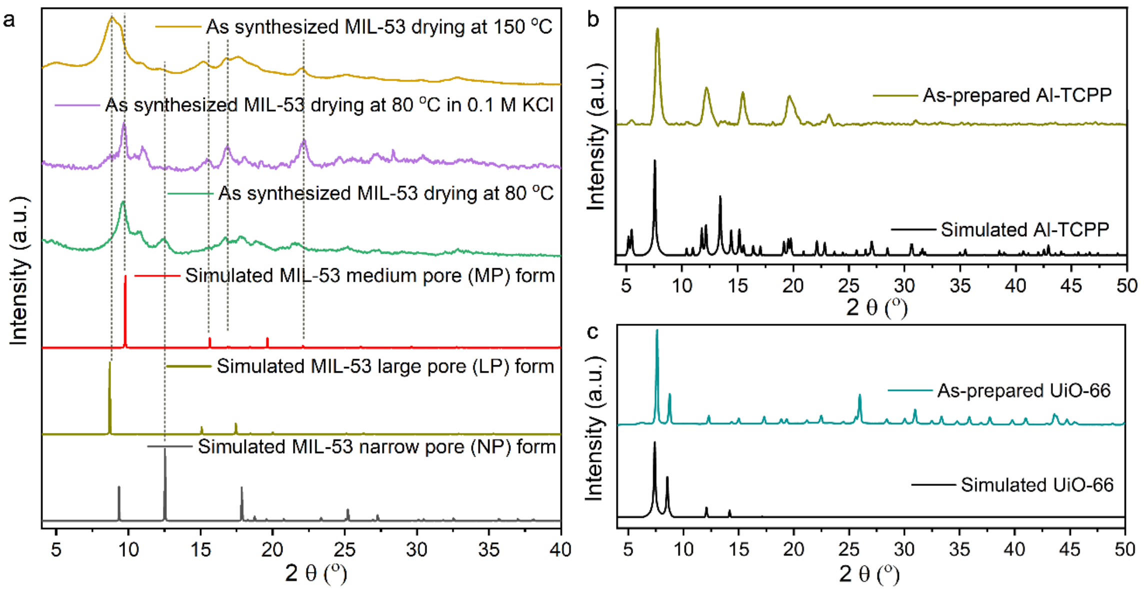

Supplementary Fig. 5. XRD patterns of MOF crystals. **a** XRD patterns of MIL-53 in the large, medium, and narrow pore forms. MIL-53 drying at 80 °C shows the MP form, and MIL-53 activated at 150 °C shows the LP form. As a comparison, MIL-53 remains the MP form after 0.1 M KCl treatment for 12 h. **b** XRD patterns of simulated and as-prepared Al-TCPP crystals. **c** XRD patterns of simulated and as-prepared UiO-66 crystals.

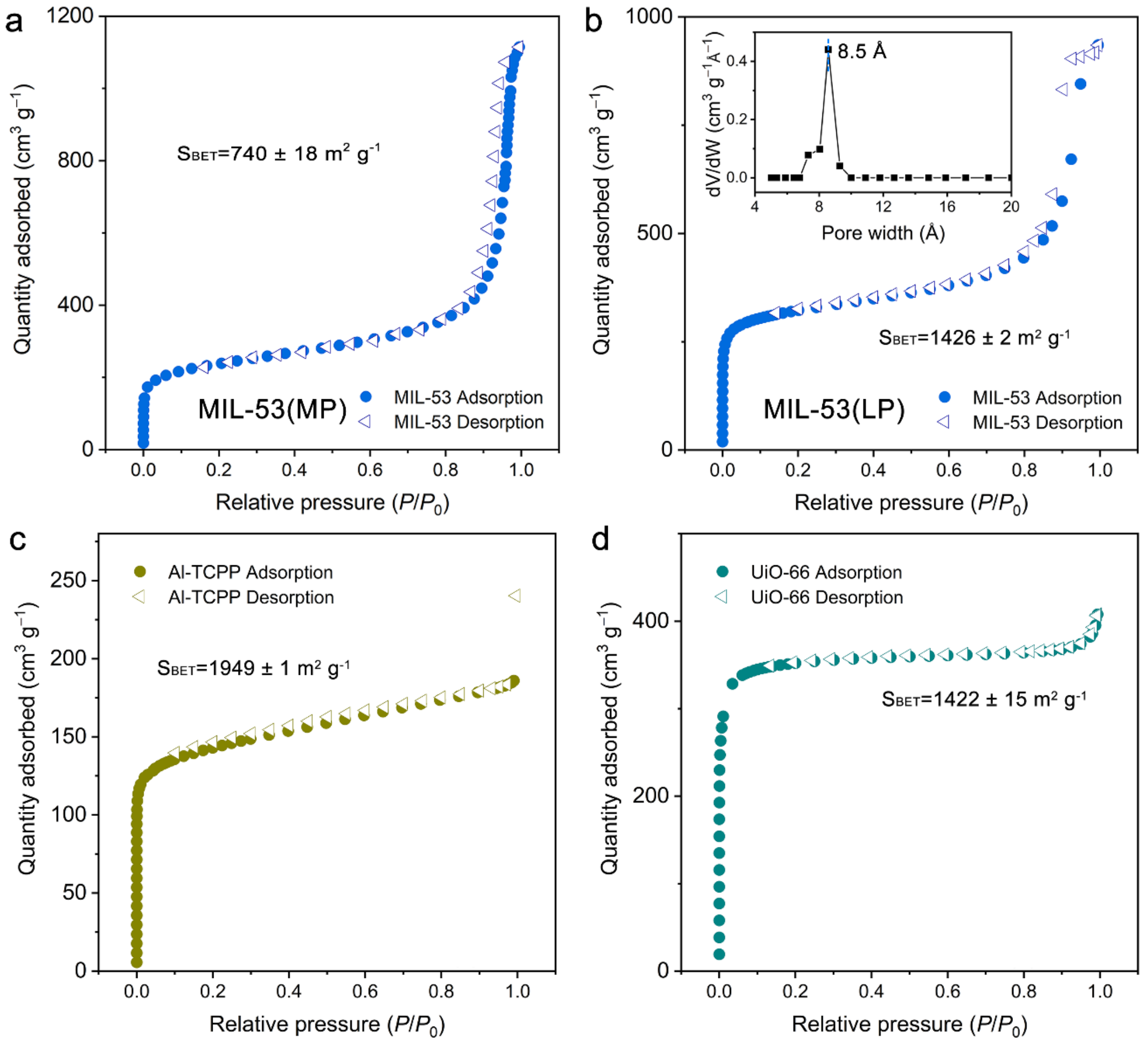

Supplementary Fig. 6. N_2 adsorption-desorption isotherms of MOF crystals. **a** N_2 adsorption-desorption isotherm of MIL-53(non-activated) in the MP form. **b** N_2 adsorption-desorption isotherm and pore size distribution of MIL-53(activated) in the LP form. **c** N_2 adsorption-desorption isotherm of Al-TCPP. **d** N_2 adsorption-desorption isotherm of UiO-66.

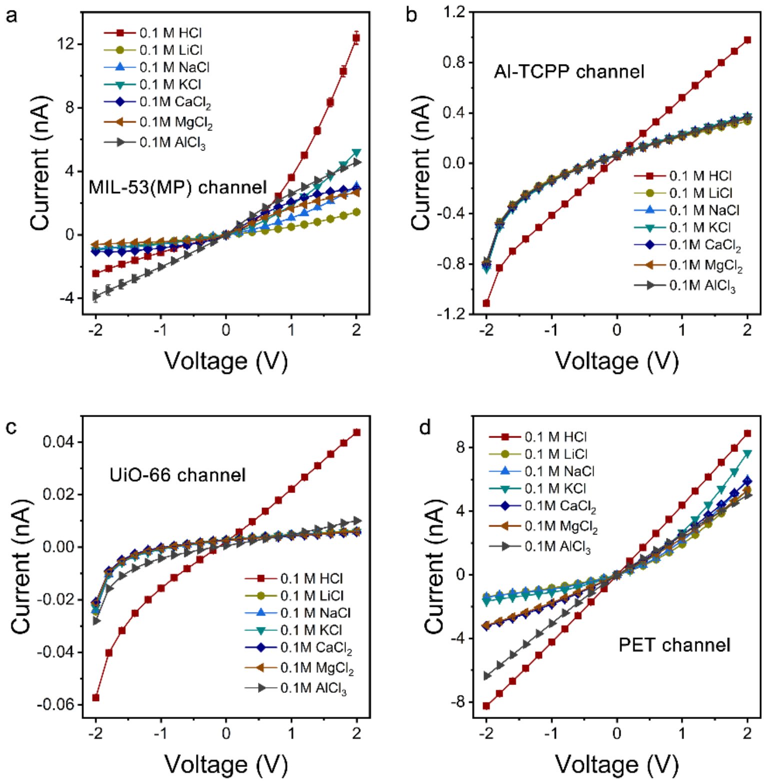

Supplementary Fig. 7. Ion transport properties in MOF channels. **a** *I-V* curves of the MIL-53(MP) channel measured in 0.1 M chloride salts. **b** *I-V* curves of the Al-TCPP channel measured in 0.1 M chloride salts. **c** *I-V* curves of the UiO-66 channel measured in 0.1 M chloride salts. **d** *I-V* curves of the PET channel measured in 0.1 M chloride salts. Error bars represent the standard deviation of three measurements of a sample.

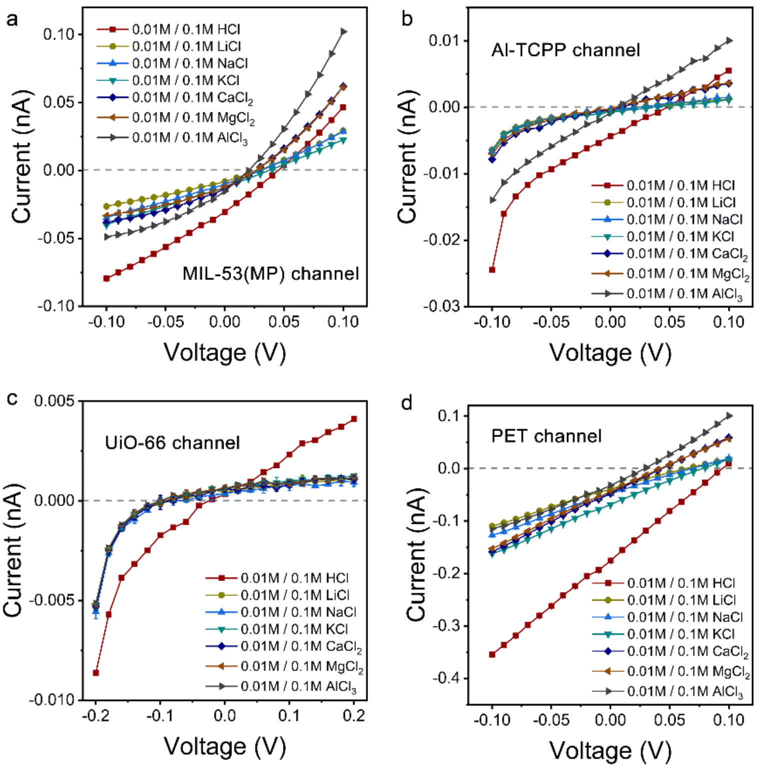

Supplementary Fig. 8. Drift-diffusion measurement of MOF channels. **a** *I-V* curves of the MIL-53(MP) channel measured in 0.1 M/0.01 M chloride salts. **b** *I-V* curves of the Al-TCPP channel measured in 0.1 M/0.01 M chloride salts. **c** *I-V* curves of the UiO-66 channel measured in 0.1 M/0.01 M chloride salts. **d** *I-V* curves of the PET channel measured in 0.1 M/0.01 M chloride salts. Error bars represent the standard deviation of three measurements of a sample.

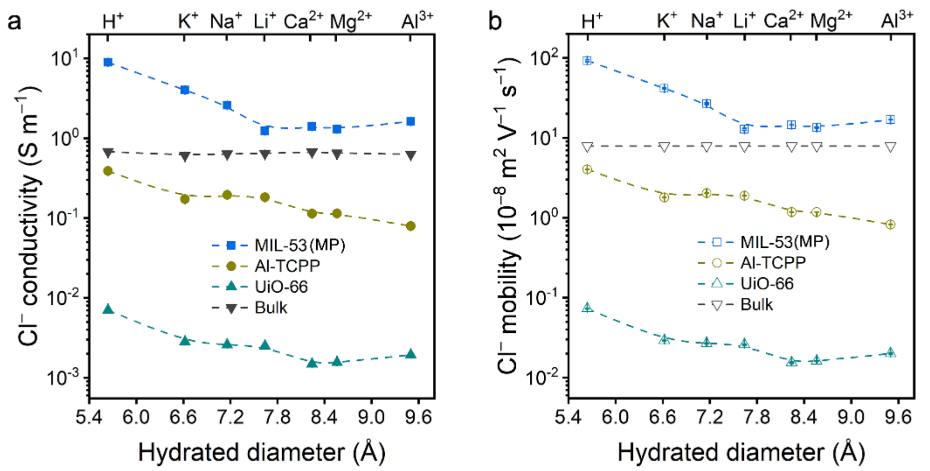

Supplementary Fig. 9. Anion transport properties in MOF channels. **a** Conductivities of Cl^- in MOF channels and bulk solution. **b** Mobilities of Cl^- in MOF channels and bulk solution. Error bars represent the standard deviation of three measurements of a sample.

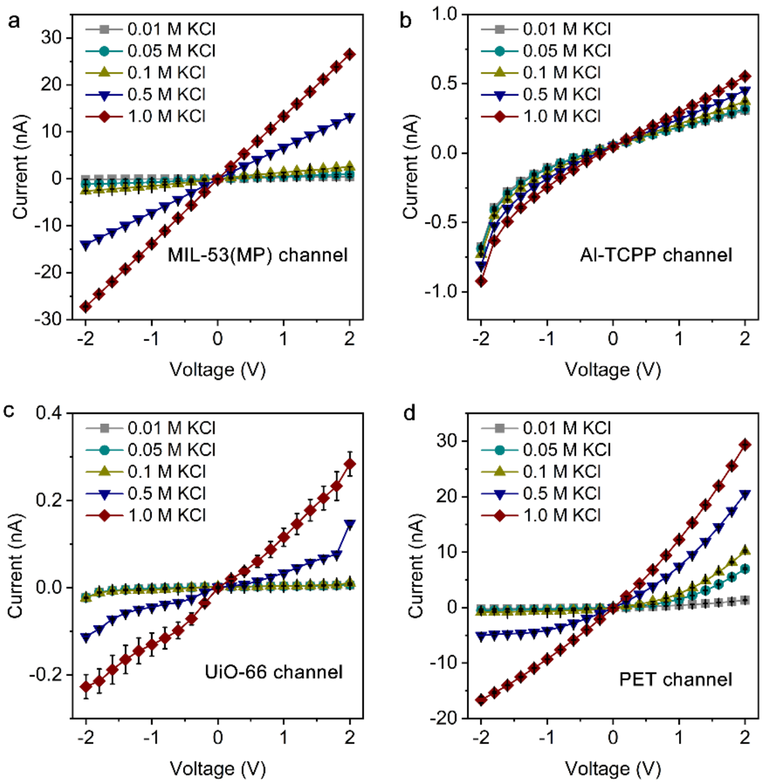

Supplementary Fig. 10. Concentration effects on ion transport properties in MOF channels. **a** *I*-*V* curves of the MIL-53(MP) channel measured in 0.01-1.0 M KCl solution. **b** *I*-*V* curves of the Al-TCPP channel measured in 0.01-1.0 M KCl solution. **c** *I*-*V* curves of the UiO-66 channel measured in 0.01-1.0 M KCl solution. **d** *I*-*V* curves of the PET channel measured in 0.01-1.0 M KCl solution. Error bars represent the standard deviation of three measurements of a sample.

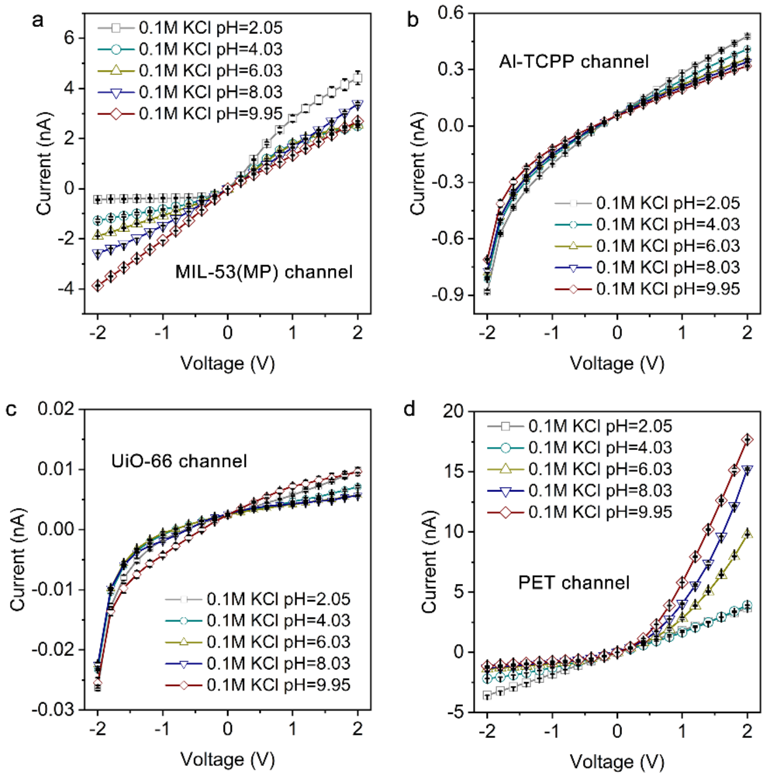

Supplementary Fig. 11. pH effects on ion transport properties in MOF channels. **a** *I-V* curves of the MIL-53(MP) channel measured in 0.1 M KCl solution with pH values from 2-10. **b** *I-V* curves of the Al-TCPP channel measured in 0.1 M KCl solution with pH values from 2-10. **c** *I-V* curves of the UiO-66 channel measured in 0.1 M KCl solution with pH values from 2-10. **d** *I-V* curves of the PET channel measured in 0.1 M KCl solution with pH values from 2-10. Error bars represent the standard deviation of three measurements of a sample.

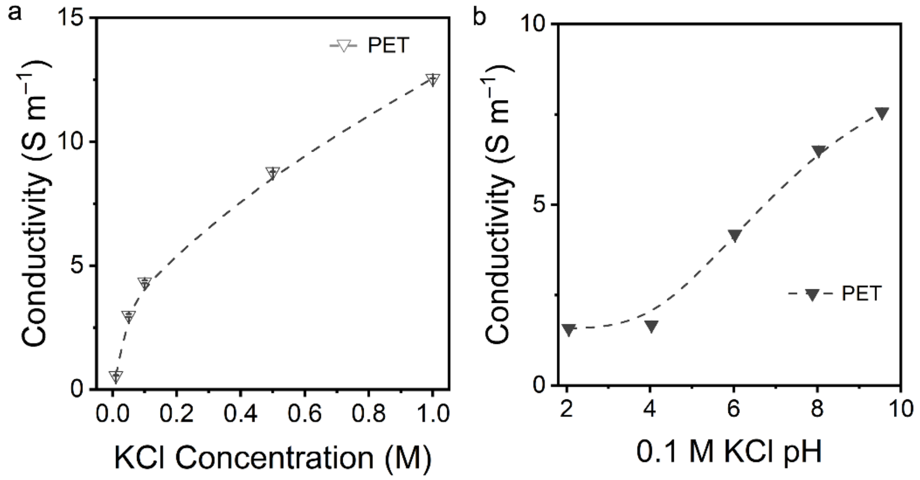

Supplementary Fig. 12. Ion transport properties in PET nanochannels. **a** Conductivities of KCl in PET channels measured in 0.01-1.0 M solution. **b** Conductivities of KCl in PET channels measured in 0.1 M solution with pH values from 2-10. Error bars represent the standard deviation of three measurements of a sample.

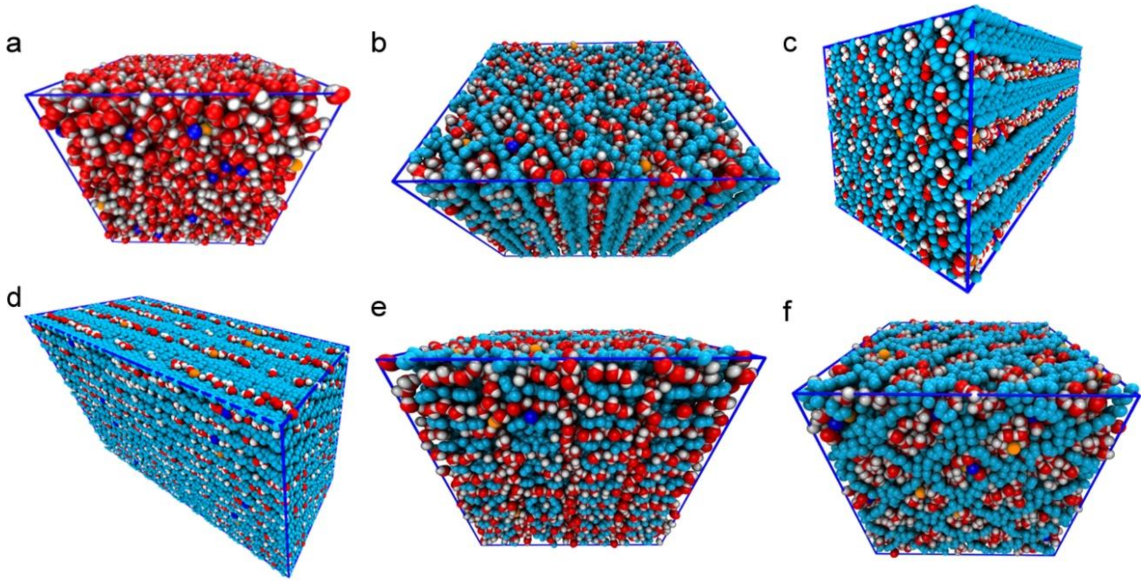

Supplementary Fig. 13. The schematics of simulation cells. **a** Bulk solution, **b** MIL-53(LP), **c** MIL-53(MP), **d** MIL-53(NP), **e** Al-TCPP, and **f** UiO-66 filled with ~1.0 M KCl solution. Note that the solid MOF wall is illustrated in blue dots.

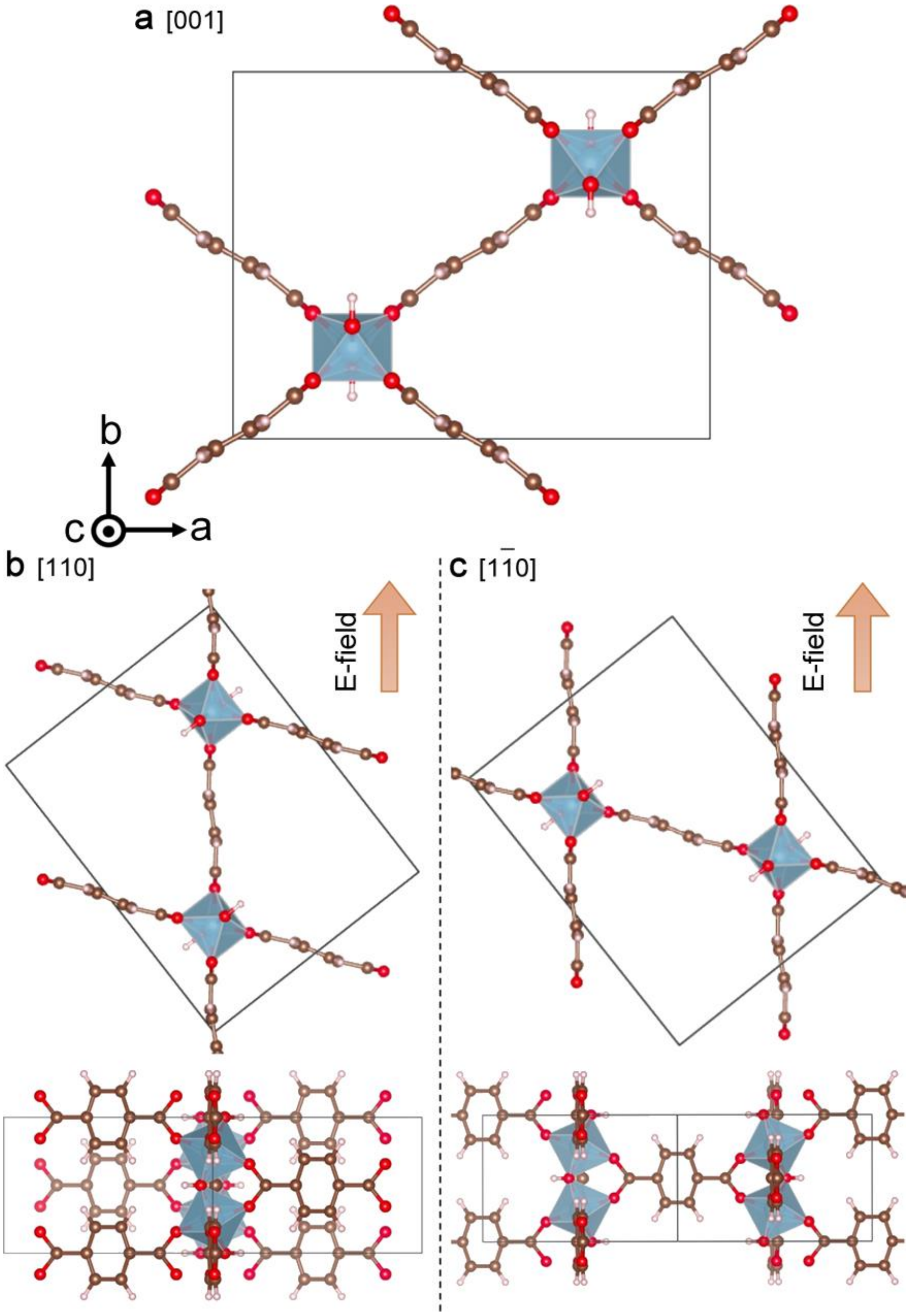

Supplementary Fig. 14. The schematic of three different *E*-field in MIL-53(LP) simulations. 1D channel in **a** [001] direction, **b** [110] direction; **c** [1$\bar{1}$0] direction. The lower parts of **b** and **c** are viewed along the *E*-field.

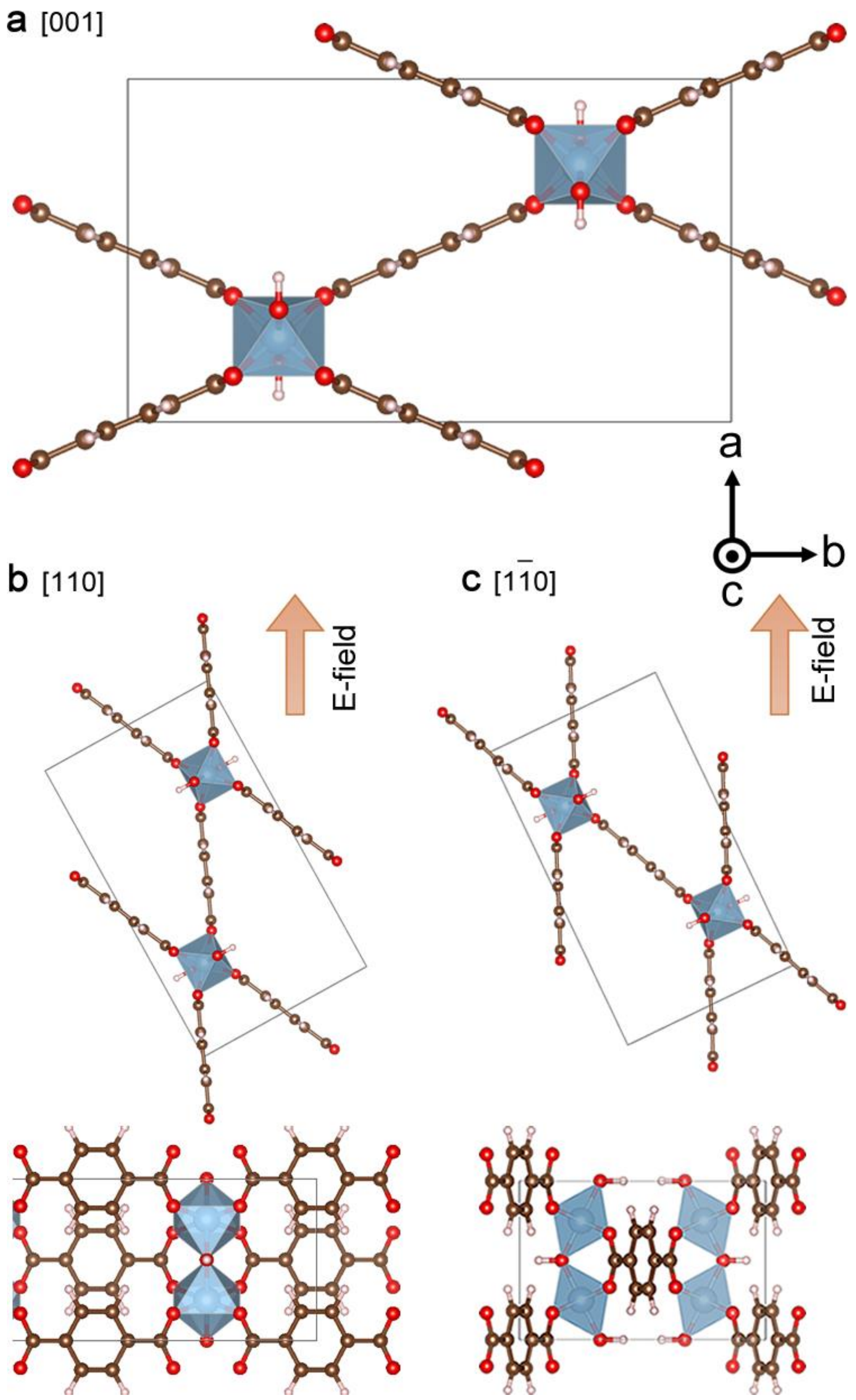

Supplementary Fig. 15. The schematic of three different *E*-field in MIL-53(MP) simulations. 1D channel in **a** [001] direction, **b** [110] direction; **c** [1$\bar{1}$0] direction. The lower parts of **b** and **c** are viewed along the *E*-field.

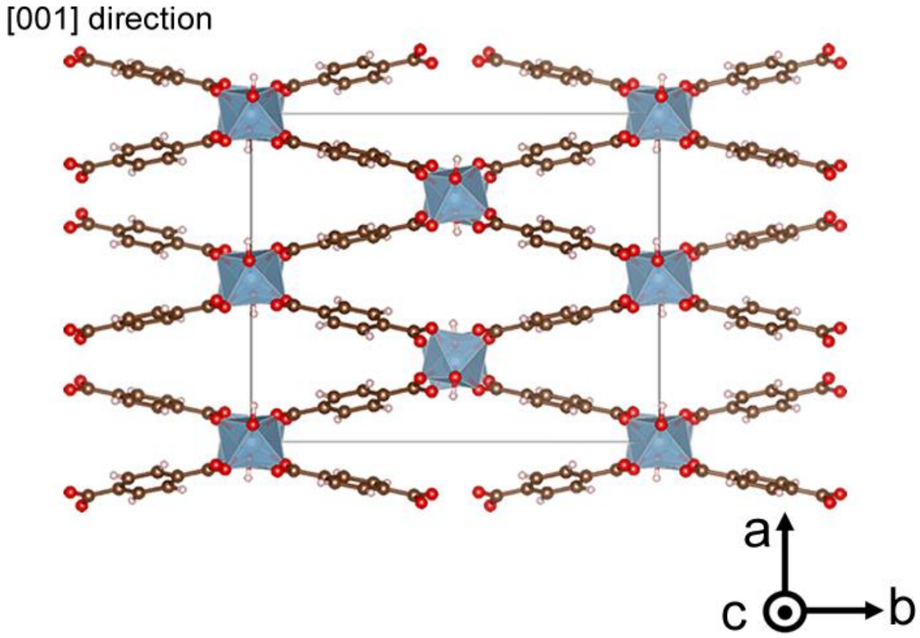

Supplementary Fig. 16. The schematic of three different *E*-field in MIL-53(NP) simulations. 1D channel in the [001] direction.

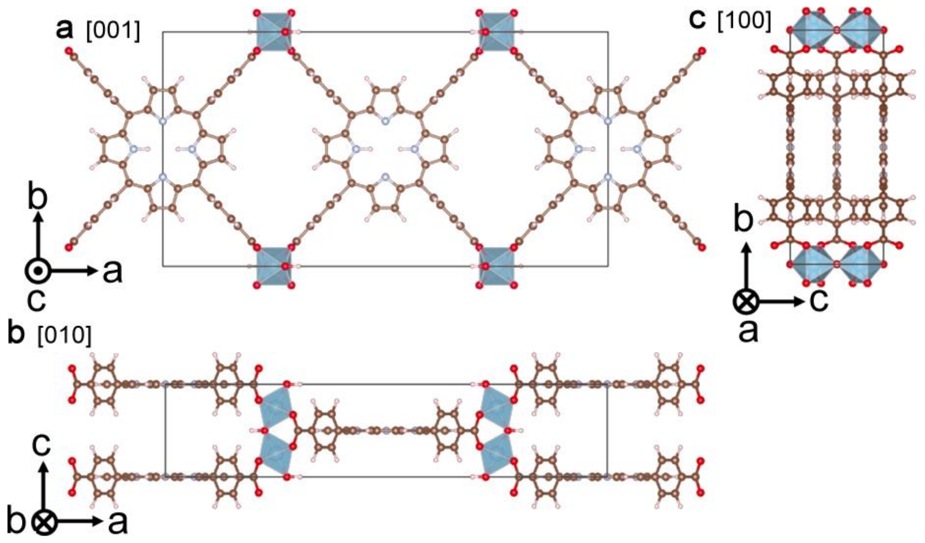

Supplementary Fig. 17. The schematics of three *E*-field in Al-TCPP simulation. 1D channel in **a** [001] direction. **b** 2D interlayer space in [010] direction. **c** 2D interlayer space in [100] direction. Note that all the *E*-fields are imposed perpendicular to the present plane.

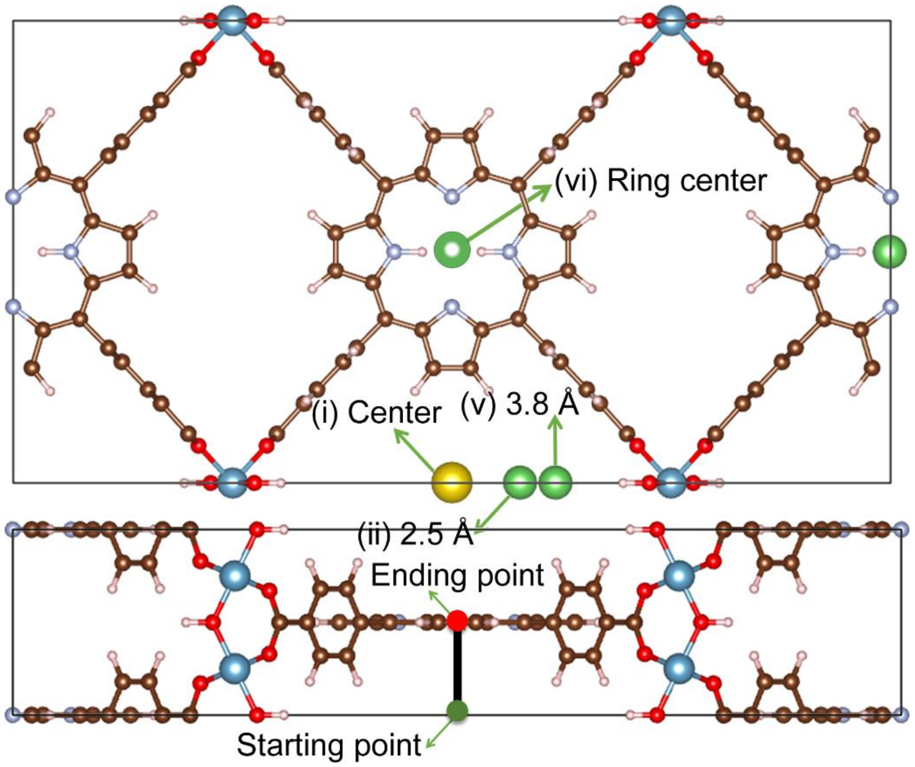

Supplementary Fig. 18. The schematics of PMF pathway in Al-TCPP for PMF calculation fixed at (i) the channel center from the green starting point to the red ending one. Another two green dots in the upper panel illustrates the minimal and maximum offsets of the pathway from channel center as (ii) 2.5 Å and (v) 3.8 Å respectively. The right middle one indicates that passing (vi) the center of porphyrin rings.

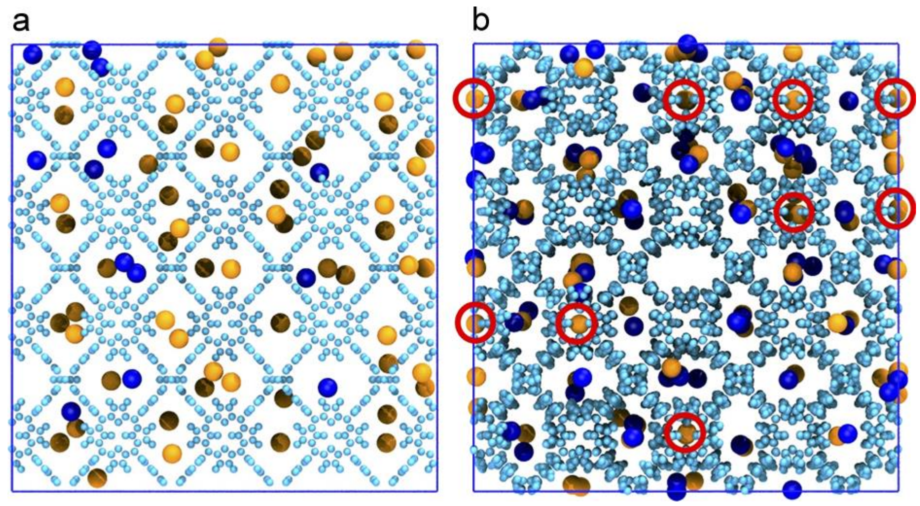

Supplementary Fig. 19. A snapshot of Al-TCPP cells. **a** The initial configuration. **b** The result after 5 ns thermal equilibrium. Note that water molecules are omitted for clarity and the orange and blue dots represent K^+ and Cl^-, respectively. The red circles represent the adsorption cations onto the center of porphyrin rings.

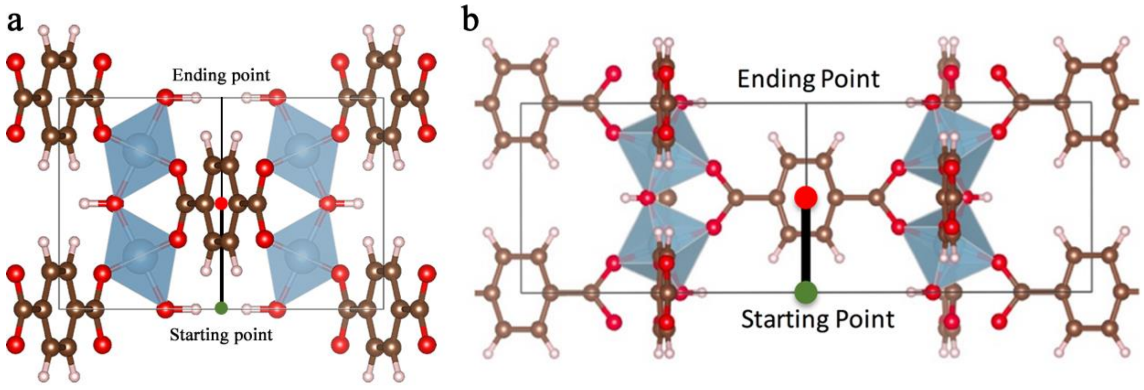

Supplementary Fig. 20. The schematic of PMF pathway in **a** MIL-53(MP), **b** MIL-53(LP), *i.e.* starting from the *xy*-plane at the green point and ending at the red one of the plane.

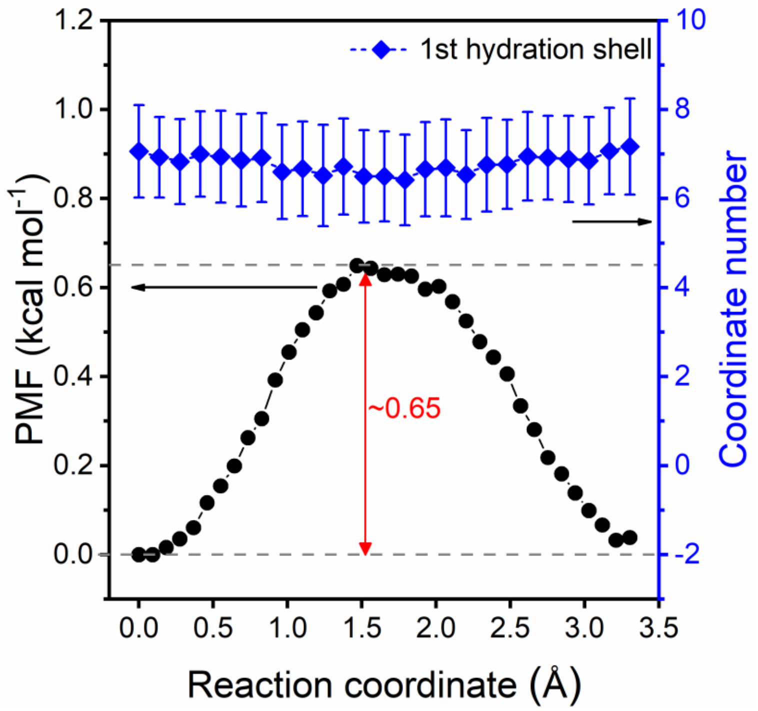

Supplementary Fig. 21. The PMF curve and first shell water hydration number of MIL-53(LP) channel. All error bars represent the standard deviation of the results by the 5 sequential time intervals.

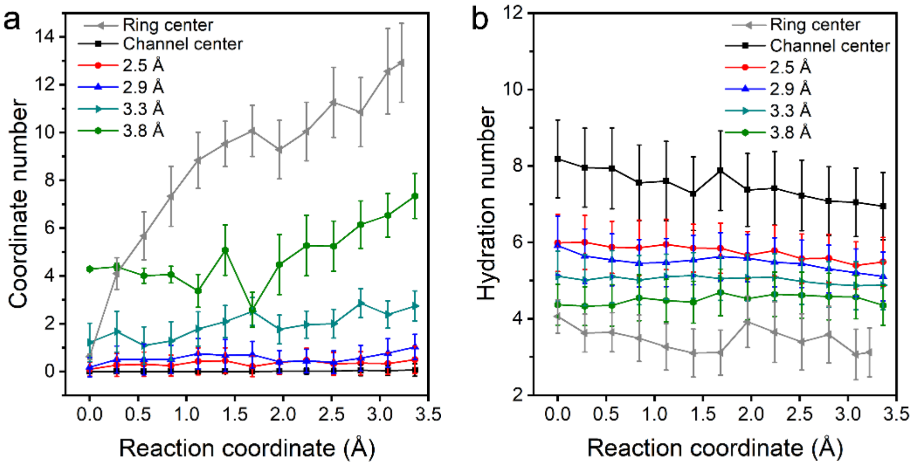

Supplementary Fig. 22. The MOF coordinate number **a** and average water hydration number **b** within the first hydration layer (*i.e.*, $r < \sim 3.6$ Å) in the different PMF sampling pathway and windows in Al-TCPP. The colored lines mark the result passing through (i) the center of 1D channel or (ii-v) some offsets from the center. The grey line is result crossing (vi) the center of the porphyrin ring. Note that the horizontal dashed line indicates the 1st hydration number in the bulk solution (*i.e.* 6.71). All error bars represent the standard deviation of the results by the 5 sequential time intervals.

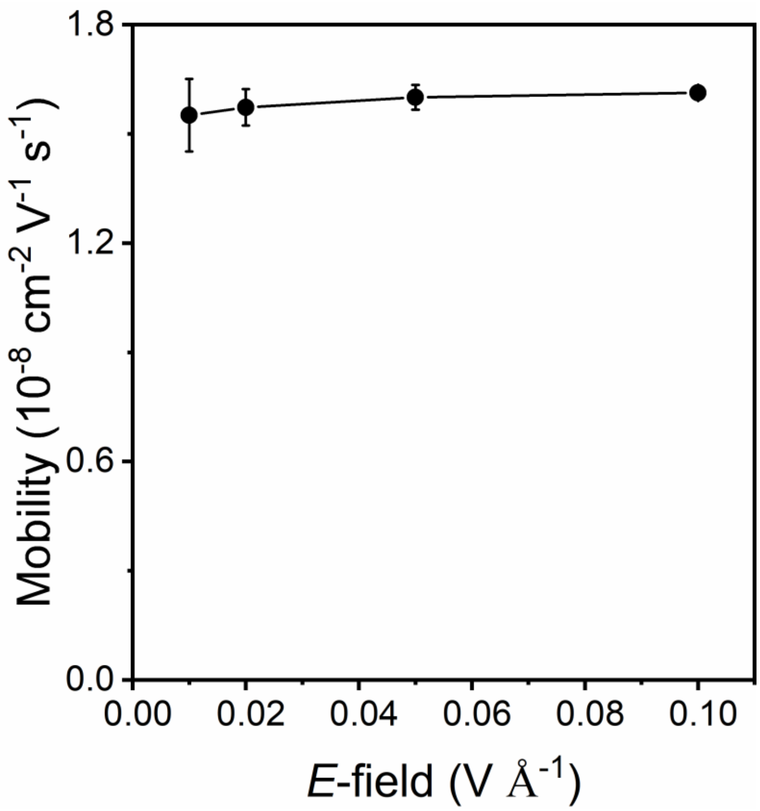

Supplementary Fig. 23. The simulated K^+ mobility in MIL-53(MP) versus the strength of *E*-field. Error bars represent the standard deviation of calculations.

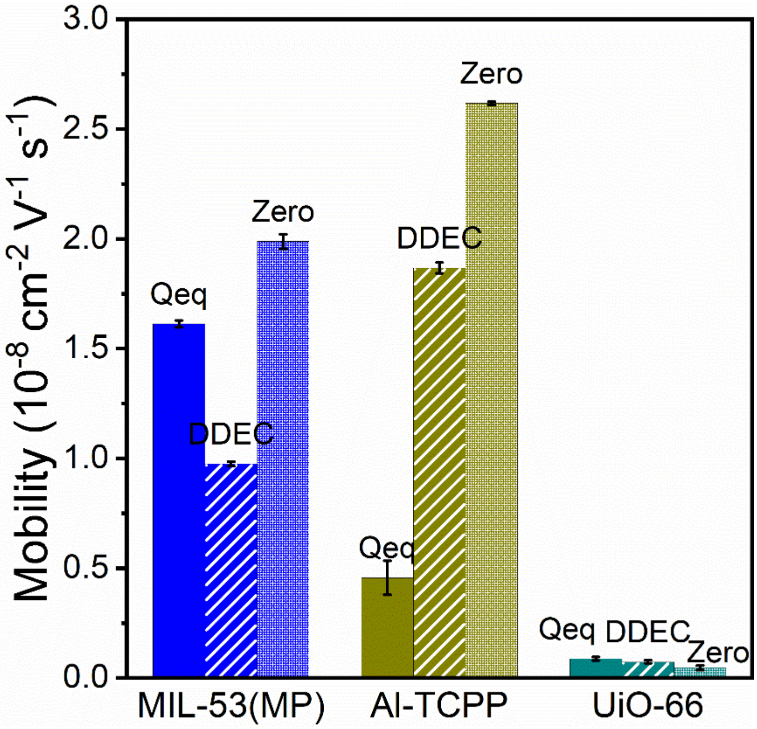

Supplementary Fig. 24. The simulated K^+ mobility in different MOF frameworks using the different partial charge assignment (Qeq, DDEC6, and Zero-charges). The mobilities along [001] direction of MIL-53(MP) by DDEC6 and Zero-charges are still two orders higher than those along [111] direction of UiO-66, indicating our conclusion is valid. But we do notice the enhanced K^+ mobility in the Al-TCPP framework by DDEC6 and Zero-charge assignments, even higher than those in the MIL-53(MP) framework. It contradicts the experimental results, which could be attributed to the incorrect charge distribution on the Al-TCPP framework. Error bars represent the standard deviation of calculations.

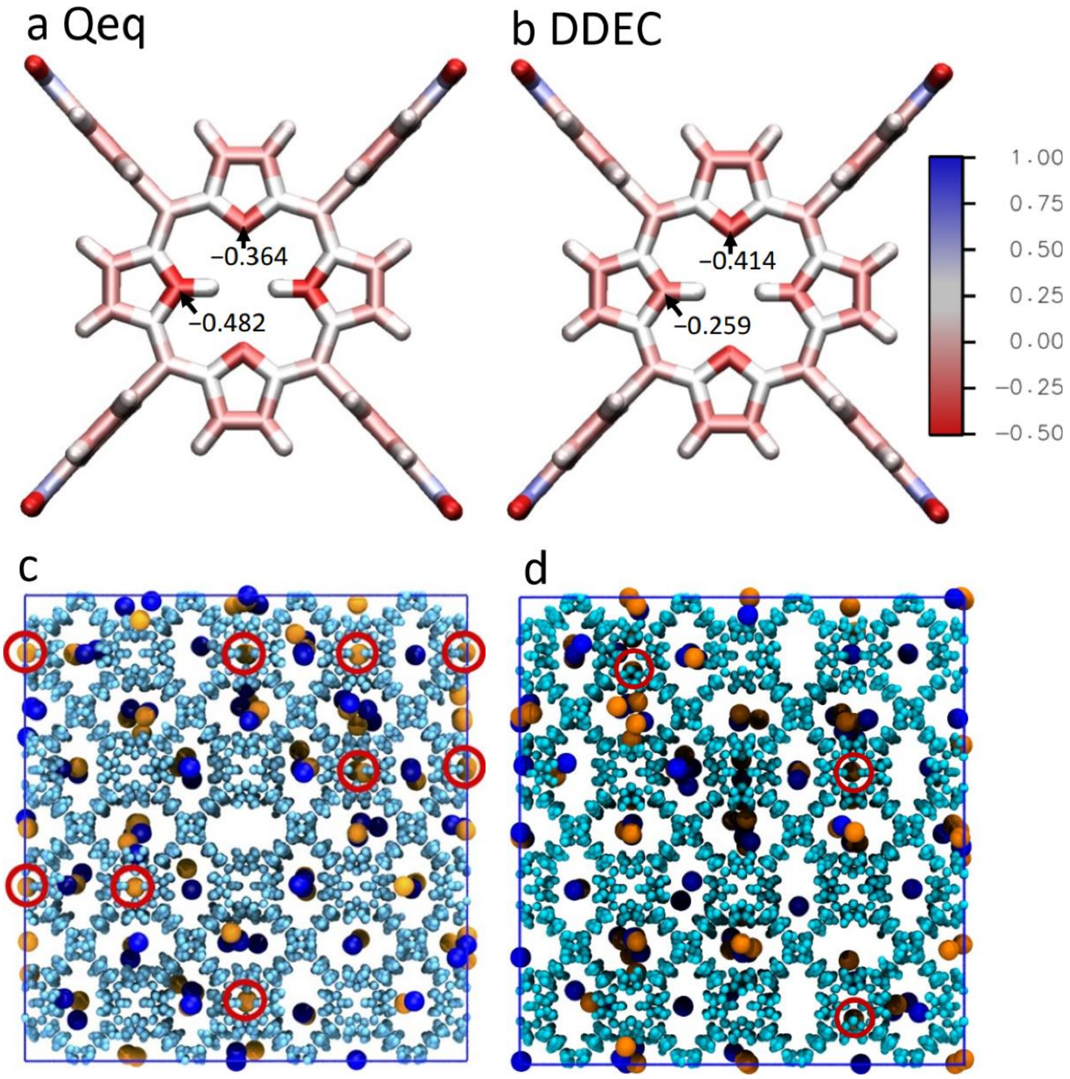

Supplementary Fig. 25. A schematic of partial charges distribution by **a** Qeq and **b** DDEC6 assignment. The equilibrium snapshots for **c** Qeq, and **d** DDEC6 Al-TCPP simulation. The magnitudes of partial charges on the porphyrin ring (the value on the ether atoms) in the DDEC6 assignment are much lower than those in the Qeq counterpart. Thus, the equilibrium snapshots show that the specific K^+ binding is significantly weakened in the DDEC6 assignment, resulting in a higher transport rate. The inconsistency with experimental results suggests that the Qeq assignment can be a better method than other methods for the MOF frameworks studied in our case.

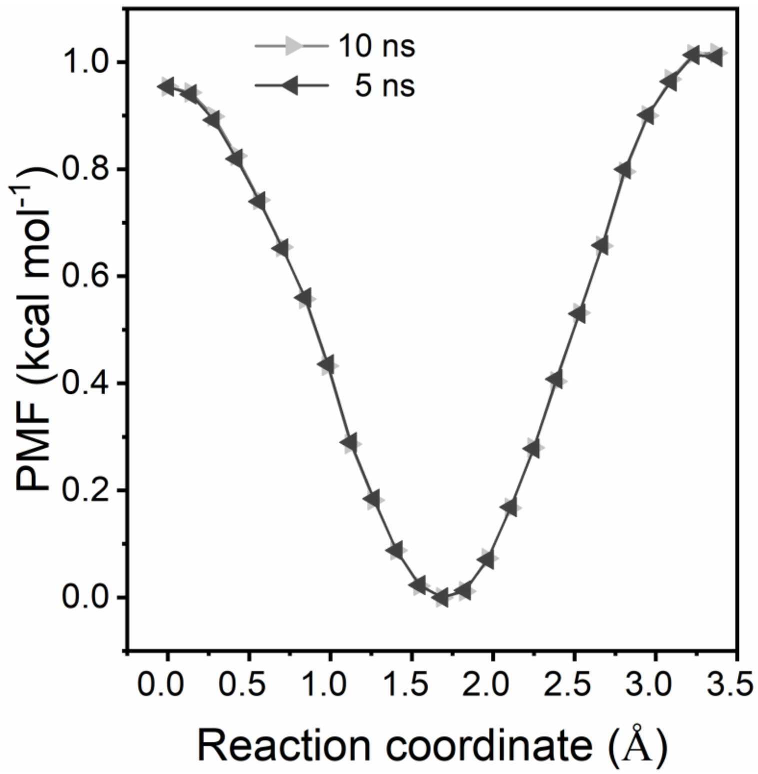

Supplementary Fig. 26. The PMF curves for the MIL-53(MP) framework along the [001] direction with different duration times (dark grey 5 ns vs grey 10 ns).

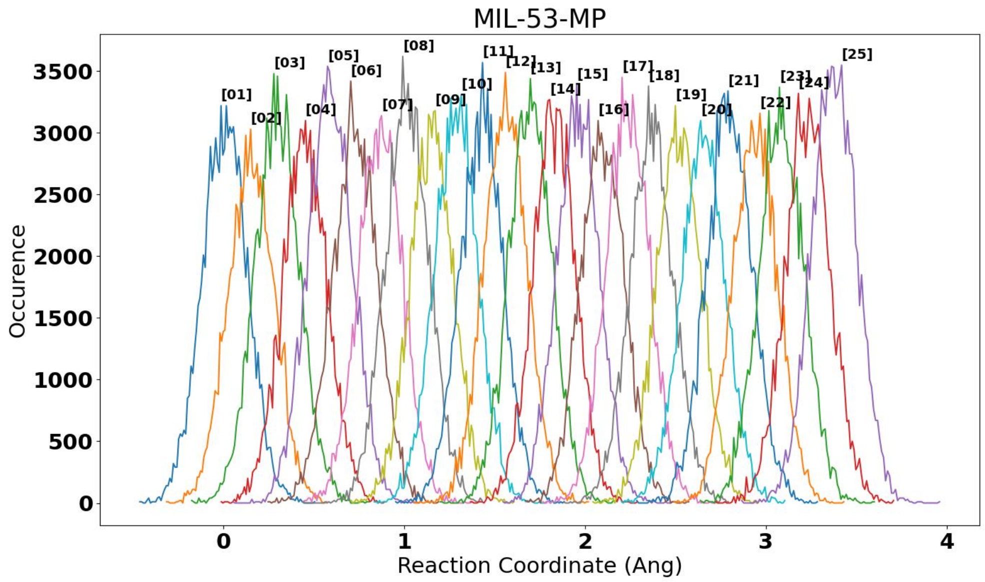

Supplementary Fig. 27. The histogram plot of umbrella samplings for MIL-53(MP) PMF simulations.

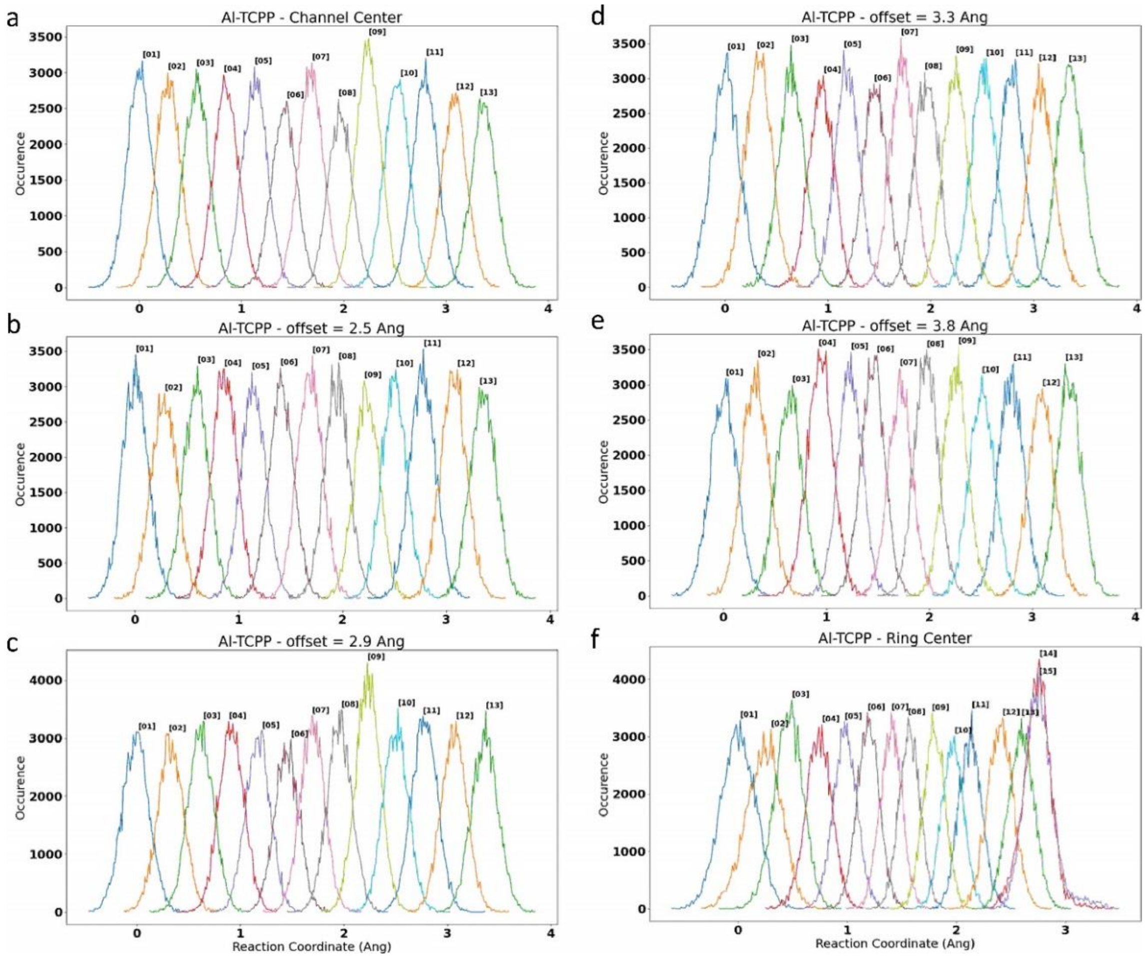

Supplementary Fig. 28. The histogram plot of umbrella samplings for Al-TCCP PMF simulations at different position: **a** Channel center, **b** Offset 2.5 Å, **c** Offset 2.9 Å, **d** Offset 3.3 Å, **e** Offset 3.8 Å, **f** Ring center.

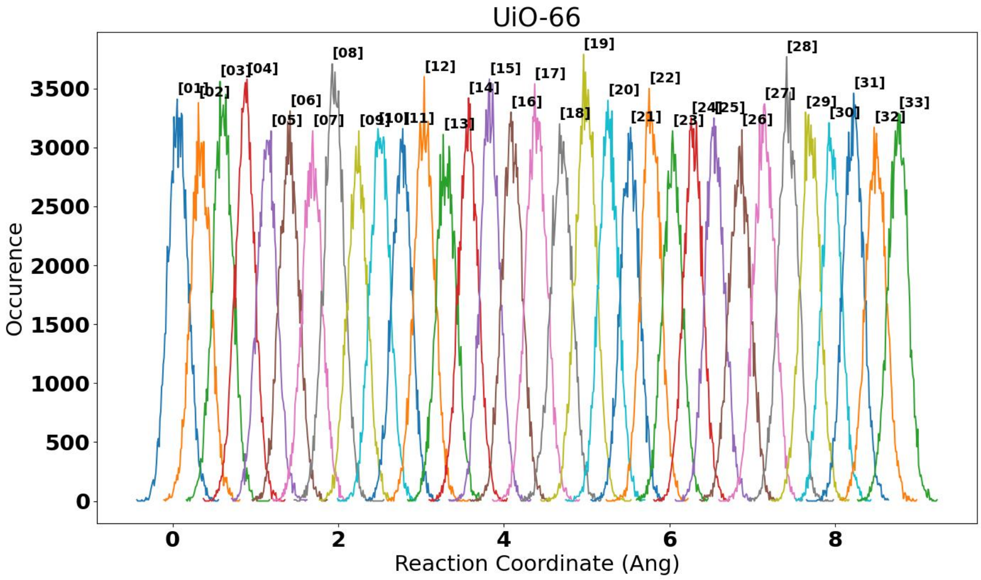

Supplementary Fig. 29. The histogram plot of umbrella sampling for UiO-66 PMF simulations.

Supplementary Table 1

Crystallographic data of MIL-53 in the large pore (LP), medium pore (MP), and narrow pore (NP) forms.

MOFs	Pore size (Å×Å)	Pore volume (cm^3 g^{-1})
MIL-53 (LP)	8.5×8.5	0.56
MIL-53 (MP)	5.2×8.9	0.35
MIL-53 (NP)	2.6×13.6	0.27

Supplementary Table 2

XPS of MIL-53 powder before and after adsorption of different chloride salts.

**Samples**	**MIL-53**		**MIL-53 in 0.1M LiCl**		**MIL-53 in 0.1M NaCl**		**MIL-53 in 0.1M KCl**		**MIL-53 in 0.1M $CaCl_2$**		**MIL-53 in 0.1M $MgCl_2$**		**MIL-53 in 0.1M AlCl3**	
	Atomic(%)/Error (%)		Atomic(%)/Error (%)		Atomic(%)/Error (%)		Atomic(%)/Error (%)		Atomic(%)/Error (%)		Atomic(%)/Error (%)		Atomic(%)/ Error (%)	
**C 1s**	60.1	0.34	62.0	0.34	61.5	0.35	59.9	0.41	59.5	0.30	59.3	0.33	60.3	0.31
**O 1s**	32.8	0.30	31.2	0.29	31.7	0.3	33.7	0.35	33.4	0.27	34.2	0.29	33.4	0.27
**Al 2p**	**7.0**	**0.14**	6.8	0.14	6.8	0.15	6.4	0.15	7.1	0.12	6.5	0.12	**6.2**	**0.13**
**Li 1s**			**0.0**	**0.00**										
**Na 1s**					**0.0**	**0.00**								
**K 2p**							**0.0**	**0.00**						
**Ca 2p**									**0.0**	**0.00**				
**Mg 1s**											**0.0**	**0.00**		

Notes: 0.05g MIL-53 powder was kept in 1.0 mL 0.1 M chloride salts for 5 h. The MOF powder was collected by filtration after washing with deionized water.

Supplementary Table 3

Crystallographic data of MIL-53(MP), Al-TCPP and UiO-66

MOFs	MIL-53(MP)	Al-TCPP	UiO-66
Pore volume ($cm^3\ g^{-1}$)	0.35	0.79	0.52
Crystal density ($g\ cm^{-3}$)	0.98	0.79	1.22

Supplementary Table 4

MD simulation parameters

	Supercell	Size (Å)	Volume (nm^3)	Water (#)	Ions (#)	Concentration (M)
Al-TCPP	2×4×10	64.39×67.86×67.22	293.50	4192	74	0.98
MIL-53(LP)	4×6×10	66.70×76.88×66.09	338.90	4704	83	0.98
MIL-53(MP)	4×6×28	72.97×62.42×188.8	860.14	8064	144	0.99
MIL-53(NP)	4×6×28	78.01×91.21×178.3†	1269.00	5376	96	0.99
UiO-66	3×3×3	62.10×62.10×62.10	239.48	3780	67	0.98
Bulk	N.A.	52.68×52.68×52.68	146.20	4704	83	0.98

The concentration of electrolyte in the last column was estimated from the water-ion ratio.

†Unlike the orthorhombic MIL-53(LP) and MIL-53(MP) structures, the crystal structure of MIL-53(NP) is monoclinic with β=104.18°.

Supplementary Table 5

PMF profiles for the MIL-53(MP) framework (Errors represent the standard deviation of calculations.).

Coordinate (Å)	Free (kcal/mol)	+/- (kcal/mol)
0.00000	0.95388	0.00691
0.14072	0.94324	0.00645
0.28123	0.89828	0.00610
0.42173	0.82482	0.00572
0.56224	0.74223	0.00532
0.70274	0.65430	0.00496
0.84325	0.55733	0.00458
0.98375	0.43221	0.00423
1.12426	0.28634	0.00385
1.26476	0.18167	0.00352
1.40526	0.08786	0.00324
1.54577	0.02180	0.00302
1.68627	0.00000	0.00293
1.82678	0.01167	0.00302
1.96728	0.07304	0.00322
2.10779	0.16739	0.00353
2.24829	0.27979	0.00389
2.38879	0.40351	0.00428
2.52930	0.53162	0.00466
2.66980	0.65669	0.00505
2.81031	0.79517	0.00547
2.95081	0.90020	0.00585
3.09132	0.96821	0.00619
3.23182	1.01488	0.00659
3.37233	1.01721	0.00695
